# Neuromodulation to the Rescue: Compensation of Temperature-Induced Breakdown of Rhythmic Motor Patterns via Extrinsic Neuromodulatory Input

**DOI:** 10.1371/journal.pbio.1002265

**Published:** 2015-09-29

**Authors:** Carola Städele, Stefanie Heigele, Wolfgang Stein

**Affiliations:** 1 Institute of Neurobiology, Ulm University, Ulm, Germany; 2 School of Biological Sciences, Illinois State University, Normal, Illinois, United States of America; The Salk Institute for Biological Studies, UNITED STATES

## Abstract

Stable rhythmic neural activity depends on the well-coordinated interplay of synaptic and cell-intrinsic conductances. Since all biophysical processes are temperature dependent, this interplay is challenged during temperature fluctuations. How the nervous system remains functional during temperature perturbations remains mostly unknown. We present a hitherto unknown mechanism of how temperature-induced changes in neural networks are compensated by changing their neuromodulatory state: activation of neuromodulatory pathways establishes a dynamic coregulation of synaptic and intrinsic conductances with opposing effects on neuronal activity when temperature changes, hence rescuing neuronal activity. Using the well-studied gastric mill pattern generator of the crab, we show that modest temperature increase can abolish rhythmic activity in isolated neural circuits due to increased leak currents in rhythm-generating neurons. Dynamic clamp-mediated addition of leak currents was sufficient to stop neuronal oscillations at low temperatures, and subtraction of additional leak currents at elevated temperatures was sufficient to rescue the rhythm. Despite the apparent sensitivity of the isolated nervous system to temperature fluctuations, the rhythm could be stabilized by activating extrinsic neuromodulatory inputs from descending projection neurons, a strategy that we indeed found to be implemented in intact animals. In the isolated nervous system, temperature compensation was achieved by stronger extrinsic neuromodulatory input from projection neurons or by augmenting projection neuron influence via bath application of the peptide cotransmitter *Cancer borealis* tachykinin-related peptide Ia (CabTRP Ia). CabTRP Ia activates the modulator-induced current I_MI_ (a nonlinear voltage-gated inward current) that effectively acted as a negative leak current and counterbalanced the temperature-induced leak to rescue neuronal oscillations. Computational modelling revealed the ability of I_MI_ to reduce detrimental leak-current influences on neuronal networks over a broad conductance range and indicated that leak and I_MI_ are closely coregulated in the biological system to enable stable motor patterns. In conclusion, these results show that temperature compensation does not need to be implemented within the network itself but can be conditionally provided by extrinsic neuromodulatory input that counterbalances temperature-induced modifications of circuit-intrinsic properties.

## Abstract 2

An electrophysiology and modelling study reveals how temperature can affect the balance of ionic conductances in neural circuits and how neuromodulators can compensate for detrimental temperature effects.

## Author Summary

All physiological processes are influenced by temperature. This is a particular problem for the nervous system, as temperature changes can disrupt the well-balanced flow of ions across the cell membrane necessary for maintaining nerve cell function. Possessing compensatory mechanisms that counterbalance detrimental temperature effects and maintain vital behaviors is especially important for poikilothermic animals, because they do not actively maintain their body temperature and can experience substantial temperature fluctuations. In this study, we analyze the mechanisms that allow the nervous system to maintain rhythmic activity over a range of different temperatures. To do so, we use the well-characterized central pattern generator of the stomatogastric nervous system of the crab that controls the motion of the gut. In this system, when experimentally isolated from the rest of the nervous system, even a small temperature increase can lead to termination of rhythmic activity due to a change in the balance of ionic conductances at elevated temperatures. However, the intact animal can compensate for these detrimental temperature effects. We demonstrate that such compensation can be achieved by restoring the balance of ionic conductance via an increase in neuromodulator release from projection neurons that control the motor circuits. We conclude that temperature compensation via neuromodulation may be a widespread phenomenon since it allows quick and flexible compensation of temperature influences on the nervous system.

## Introduction

Maintaining neural function at different temperatures is a particularly difficult challenge for the nervous system since all biophysical processes are temperature dependent. General knowledge about how this task is achieved remains rather limited, in particular since all biological processes, including those that govern signal transduction and neuronal excitability, vary substantially in their response to temperature [1]. Central pattern generators (CPGs) are a class of neural networks that generate rhythmic activity patterns. CPG activity has to be particularly resilient against perturbations because many CPGs drive vital behaviors such as respiration, swallowing, and locomotion. CPG activity depends on the coordinated interplay between synaptic and cell-intrinsic ionic conductances [2]. Many conductance combinations can give rise to rhythmicity, allowing networks to individually vary in conductance levels while remaining within the permissive conductance space for rhythmic activity. It has been suggested that compensation of temperature perturbations may be achieved by keeping conductance levels within this permissive range via a balanced coregulation of cellular and synaptic properties that result in opposing effects on network output. For example, phase constancy in the pyloric rhythm of crabs over a wide temperature range is accompanied by a balanced change of two opposing conductances (I_h_ and I_A_; [3,4]). In *Aplysia*, release of a neuromodulator that modulates muscle contraction drops 20-fold at higher temperatures, but this drop is partially counterbalanced by an increase in modulator efficacy [5]. More recent studies indicate that neuromodulators may contribute to temperature compensation: Thuma et al. [6] show that dopamine modulation can restore muscle force after temperature-induced loss of muscle contractions.

**Fig 1:**
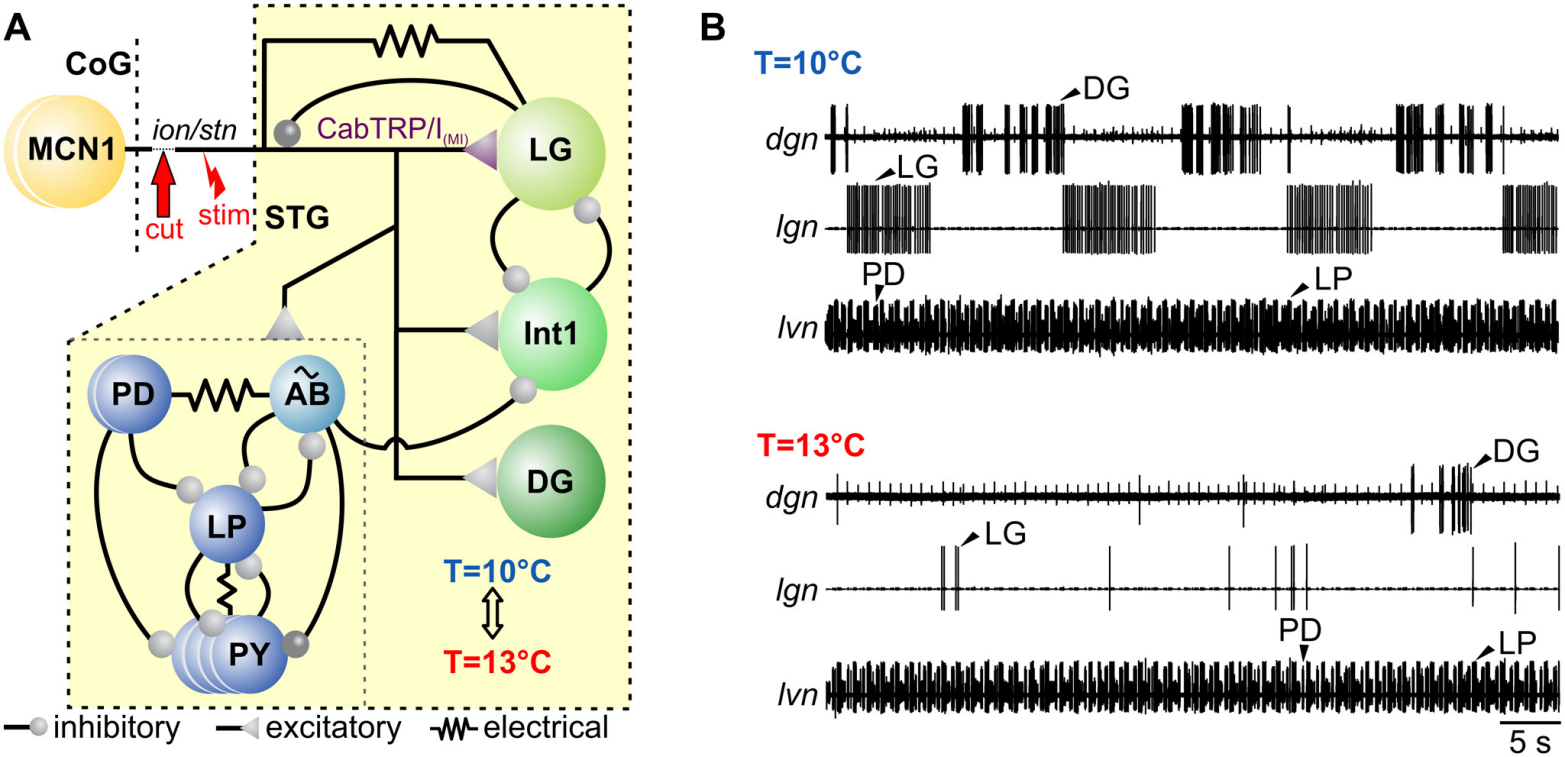
Pyloric and gastric mill networks in the *Cancer borealis* STG respond differently to temperature perturbation. (A) Main connectivity in the STG and innervation by the CoG projection neuron MCN1 (yellow). Blue circles represent pyloric neurons, and green circles represent gastric mill neurons. The pyloric and gastric mill CPGs receive excitatory input from MCN1, which innervates the STG via the *ion* and *stn*. MCN1 elicits a distinct version of the gastric mill rhythm that includes rhythmic bursting in LG, driven by the release of the peptide CabTRP Ia, electrical coupling between the MCN1 axon terminal and LG, and presynaptic inhibition of the MCN1 terminal [12]. CabTRP Ia activates the modulator-activated inward current I_MI_ [14]. Bursting in LG also requires reciprocal inhibition with Int1. The pyloric circuit is pacemaker driven (AB) and modulated by MCN1. In most experiments, MCN1 influence was controlled by decentralizing the STG (arrow) and extracellular stimulation of the MCN1 axon in the remaining *ion* (“stim”). The yellow area indicates that temperature perturbations only affected the STG. (B) Example extracellular nerve recordings showing the spontaneous pyloric and gastric mill rhythms at low (T = 10°C) and elevated temperature (T = 13°C). Three extracellular recordings are shown. Top: dorsal gastric nerve *dgn* showing the activity of the dorsal gastric (DG) neuron. DG is a functional antagonist of LG. Middle: lateral gastric nerve *lgn*, showing the activity of LG. Bottom: lateral ventricular nerve *lvn*, showing the pyloric rhythm. The pyloric rhythm is triphasic and consists of the alternating activities of the PD neurons and the lateral pyloric (LP) and pyloric constrictor (PY) neurons. At 13°C, LG and DG activities cease, and the gastric mill rhythm terminates. Recordings are from the same preparation.

This study tests the hypothesis that temperature compensation is conditional and under control of extrinsic neuromodulatory input fibers eliciting compensatory changes that oppose temperature-induced changes in intrinsic conductance levels. For this, we use the well-characterized pyloric (filtering of food) and gastric mill (chewing) CPGs in the crustacean stomatogastric ganglion (STG; Fig 1A; [7,8]), which, like most CPGs, are modulated by well-regulated extrinsic neuromodulatory pathways [9]. The triphasic pyloric motor pattern is driven by a three-neuron pacemaker ensemble (the single anterior burster [AB] and two pyloric dilator [PD] neurons) that allows it to be continuously active with and without modulatory input [10]. The phase relationship of the pyloric rhythm is maintained constant over a broad temperature range (7°C to 31°C; [3,4]). In contrast, the gastric mill rhythm is two-phasic, episodic, and driven by half-center oscillations of Interneuron 1 (Int1) and the lateral gastric (LG) neuron (Fig 1A; [11,12]). Rhythmic gastric mill activity requires modulatory input from descending projection neurons in the commissural ganglia (CoG; [8,13]). Modulatory commissural neuron 1 (MCN1), for example, mediates various sensory responses and elicits a robust gastric mill rhythm [7,12].

We provide direct evidence that temperature compensation in the gastric mill network depends on a balanced change of circuit-intrinsic properties with opposing function that are regulated by descending modulatory input from MCN1. Modest temperature increase by 3°C led to a cessation of CPG activity that was caused by a concomitant increase in leak currents in LG. Dynamic clamp-mediated subtraction of the leak was sufficient to rescue the rhythm and achieve temperature compensation. Compensation was also achieved by stronger extrinsic neuromodulatory input from MCN1 or by augmenting MCN1’s influence with bath application of MCN1’s peptide cotransmitter *C*. *borealis* tachykinin-related peptide Ia (CabTRP Ia) [15,16]. CabTRP Ia release from MCN1 activates a modulator-induced current (I_MI_ [17]) in LG, and we show that this current effectively acts as a negative leak current to counterbalance the detrimental effects of the leak increase. Thus, temperature compensation is under extrinsic neuromodulatory control, allowing conditional compensation of rapid temperature influences.

## Results

### Temperature Increase Terminates the Gastric Mill Rhythm

To test the role of circuit extrinsic neuromodulatory inputs for counterbalancing temperature-induced changes on CPG activity, we altered the temperature of the STG motor circuits but kept the CoGs at a constant temperature. The CoGs contain descending projection neurons that provide extrinsic modulatory input to the STG circuits. This approach is fundamentally different from previous studies [3,4] in which extrinsic neuromodulatory input from CoG projection neurons as well as STG motor circuits were affected by temperature changes. Here, we thermally isolated the STG circuits from the rest of the nervous system by building a petroleum jelly well around the STG. Extrinsic input fibers such as the descending CoG projection neurons remained mostly unaffected by these temperature changes. However, the axon terminals of some projection neurons have local synaptic interactions within the STG [11] and may show ectopic spike initiation. Temperature effects on these interactions were not investigated in this study. CPG activity in the STG was recorded extracellularly on three different motor nerves containing the axons of pyloric and gastric neurons (pyloric: PD, LP, and PY on the *lvn*; gastric: LG on the *lgn* and DG on the *dgn*). We found that a moderate temperature increase from 10°C to 13°C had distinct effects on the two rhythms (Fig 1B): the pyloric rhythm was resilient to temperature changes and continued its regular activity, while the spontaneous gastric mill rhythm terminated, as can be seen by the sporadic and nonrhythmic activity of the gastric mill neurons LG and DG on the *lgn* and *dgn* (Fig 1B).

The pyloric rhythm had previously been shown to be “resistant” against temperature perturbations in vivo and in vitro (although for much larger temperature ranges, up to 26°C or more; [3,18]) in that the phase relationship of the pyloric neurons remains constant while cycle period decreases. In all previous studies, however, temperature affected CPG as well as its input fibers, making it unclear whether extrinsic inputs from other parts of the nervous system are necessary to maintain the rhythm or not. Despite the fact that in our experiments the temperature perturbation exclusively affected the STG, we found similar results for the pyloric rhythm as described previously. In none of our experiments did the pyloric rhythm cease or show any obvious change from its canonical pattern (see S1 Fig). In fact, this was true even when we increased the temperature up to 19°C (*N* = 6). To conclude, our results predict that the broad temperature range of the pyloric rhythm is likely to be intrinsic to the STG circuit and that the permissive temperature range of this rhythm is independent of temperature effects on other areas of the nervous system, such as the CoGs.

**Fig 2:**
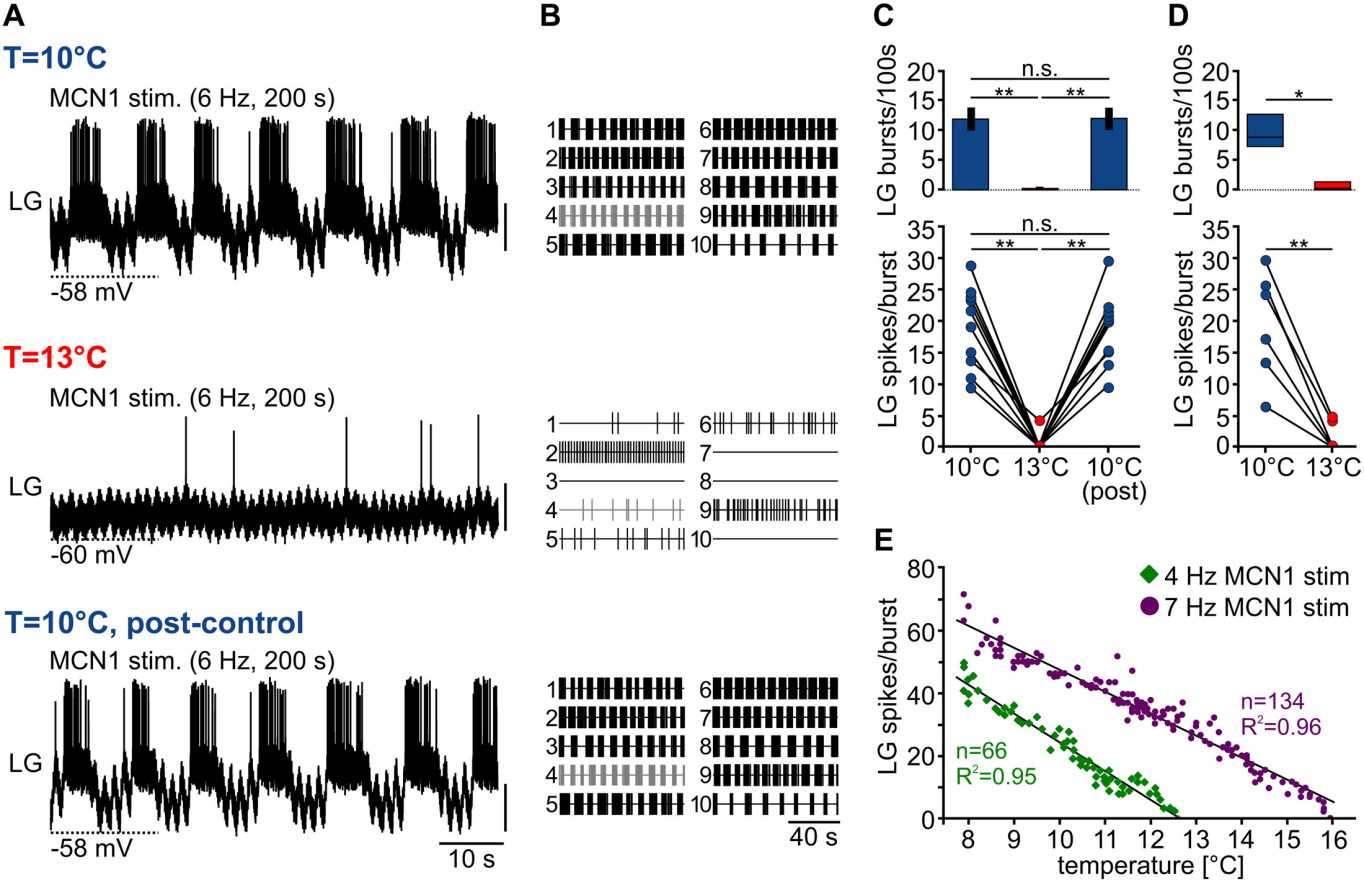
The MCN1 gastric mill rhythm terminates at elevated temperature. (A) Intracellular recording of LG during continuous extracellular MCN1 stimulation with 6 Hz at 10°C (top), 13°C (middle), and 10°C (bottom, post-control). LG was rhythmically active at 10°C (top). Rhythmic LG activity ceased at 13°C (middle) but could be restored by changing the temperature back to 10°C (bottom). Vertical scale bars, 10 mV. (B) Representation of LG spike activity for all preparations tested (*N* = 10) at 10°C (top), 13°C (middle), and 10°C post-control (bottom). Each trace shows 100 s during continuous MCN1 stimulation, with each vertical line representing an action potential in LG. Grey traces (trace 4) correspond to recordings shown in A. (C) Analysis of number of LG bursts/100 s (top) and LG spikes/burst (bottom) for all preparations tested (*N* = 10). Temperature was changed at 1°C/min. One-way repeated measures analysis of variance (RM ANOVA), F(2,18) = 280.503 (top), and F(2,18) = 76.963 (bottom), *p* < 0.001, Holm-Sidak post hoc test with *p* < 0.01 significance level. (D) Number of LG bursts/100 s (top) and LG spikes/burst (bottom) with slow temperature change (1°C/h). *N* = 6, Wilcoxon signed rank test, Z = 2.264, *p* = 0.031 for LG bursts/100 s and paired *t*-test, *p* < 0.01 for LG spikes/burst. (E) Change in number of LG spikes/burst plotted as a function of temperature from 8°C to 16°C during continuous temperature increase (~1°C/min). The different colors represent the LG response to two different MCN1 stimulation frequencies (4 Hz and 7 Hz). Stimulations were performed in the same preparation. Regression slope 4 Hz = −9.81*LG spikes/burst; 7 Hz = −7.52*LG spikes/burst, slopes significantly different from 0 with *p* < 0.001.

The gastric mill rhythm depends on the activity of upstream modulatory projection neurons in the CoGs [7,8]. About 20 CoG projection neurons innervate the STG via the unilateral stomatogastric nerve (*stn*, see Fig 1A, [19]). Sensory input like olfactory or mechanosensory stimuli [20–22] as well as sensory feedback from proprioceptors [20,23,24] activates CoG projection neurons and elicits gastric mill activity. Even individual projection neurons can start the gastric mill rhythm in vitro [12] and in vivo [25]. One particularly well-characterized projection neuron is MCN1 [11,12,25], a bilaterally symmetric neuron in each CoG with axonal projections to the STG (Fig 1A). To study the mechanism of the temperature-induced breakdown of the gastric mill rhythm, we first decentralized the nervous system to remove the influence of all CoG projection neurons. To initiate a gastric mill rhythm, we then stimulated MCN1 tonically with the lowest frequency eliciting a gastric mill rhythm at 10°C (= threshold frequency, see Materials and Methods). MCN1 was activated by extracellular stimulation of the inferior oesophageal nerve (*ion*), which contains the axons of only two projection neurons, MCN1 and MCN5. MCN1 has the lower stimulation threshold of the two and can thus be activated selectively [11,12]. For the analysis, we focused on LG since this neuron is part of the core pattern generator of the gastric mill rhythm and has a strong influence on all other gastric mill neurons: if spiking is prevented in LG, the gastric mill rhythm stops [11]. Similarly to the spontaneous gastric mill rhythm, LG activity was rhythmic at 10°C during MCN1 stimulation but became substantially reduced and irregular at 13°C (Fig 2A). In four of ten experiments, LG spiking ceased completely (Fig 2B). This effect was reversible, i.e., the rhythm returned to its original regularity and strength when temperature was decreased back to 10°C.

To quantify temperature effects on LG firing rate, we counted the number of LG bursts in 100 s and the number of LG spikes per burst (see Materials and Methods). We found that at 10°C, LG showed rather regularly spaced action potentials during the bursts followed by relatively long interburst intervals (Fig 2B). In contrast, at 13°C LG was either not active or its firing was erratic or tonic. LG activity was never rhythmic at this temperature (Fig 2B). Concurrently, the number of LG bursts, as well as the number of LG spikes per burst, dropped significantly (Fig 2C). When temperature was returned to 10°C, in all preparations rhythmicity was recovered, and the number of LG bursts and spikes per burst returned to control values (Fig 2C).

In these experiments, temperature was changed by ~1°C/min, and measurements were taken at 10°C and 13°C. Given that physiological temperature changes might occur over a longer time period than in our experiments, we performed an additional set of experiments in which we slowly changed the temperature (1°C/h). The goal of this set of experiments was to examine if homeostatic processes exist that compensate (slow) temperature perturbations. Specifically, we kept the temperature at 10°C for 1 h, then slowly increased temperature by ~1°C/h, and recorded LG activity at 10°C and 13°C. However, we found no obvious difference as compared to the faster temperature ramps used earlier: again, there was a significant decrease in the number of LG bursts and spikes per burst (Fig 2D), demonstrating that the cessation of LG rhythmicity is not counterbalanced by homeostatic processes in vitro.

Next, we tested if the termination of the rhythm occurs abruptly or in a graded fashion. Like in previous experiments, MCN1 stimulation frequency was determined at 10°C. Stimulation was then stopped, and the temperature was lowered to 8°C. Stimulation was then restarted to elicit rhythmicity, and temperature was continuously increased by 1°C/min until LG firing ceased. We found that the number of LG spikes per burst continuously decreased linearly as temperature was increased (Fig 2E, green trace). In the example shown, all burst activity stopped as temperature reached 12.5°C. The linear decrease in LG spike activity was consistent across preparations, and on average LG bursting stopped at 12.7 ± 0.2°C (*N* = 5). Additionally, we tested the permissive temperature range for normal operation of this system by performing experiments with a broader temperature range (8°C to 16°C, *N* = 5). In these experiments, we increased the MCN1 stimulation frequency (175% threshold frequency) to facilitate LG rhythmicity at temperatures above 13°C. Again, we found that the number of LG spikes per burst continuously decreased in a highly linear fashion as temperature was increased (Fig 2E, purple trace). In the example shown, all burst activity stopped as temperature reached 15.8°C. On average, this happened at 15.9 ± 0.4°C (*N* = 5). The linear response to temperature changes indicates that the gastric mill CPG is unable to compensate moderate temperature influences. The permissive temperature range, however, increased with higher MCN1 stimulation frequencies. In summary, even a moderate temperature increase led to a consistent disruption of the gastric mill rhythm, which was in stark contrast to the robust behavior of the pyloric rhythm.

### Membrane Responses Are Diminished at Higher Temperatures

**Fig 3:**
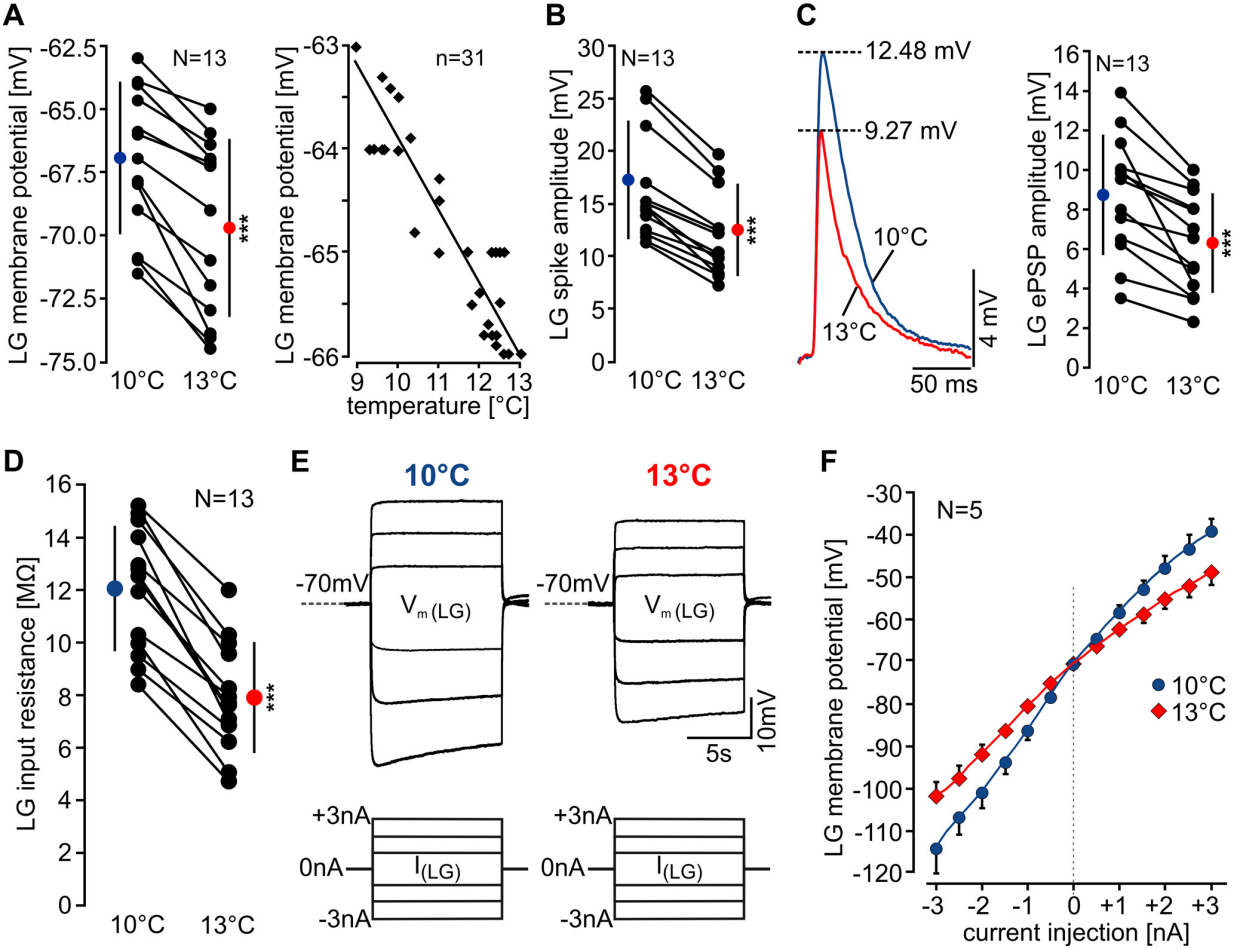
LG membrane properties are affected by temperature change. (A) Left: LG resting membrane potential at 10°C and 13°C for all tested preparations. Black circles represent individual experiments; colored circles are means ± standard deviation (SD). LG membrane potential hyperpolarized significantly at 13°C (*N* = 13, paired *t*-test, *p* < 0.001). Right: Decrease in LG resting membrane potential as a function of temperature from 9°C to 13°C during continuous temperature increase (1°C/min) in one preparation. (B) LG spike amplitude at 10°C and 13°C for all tested preparations. LG spike amplitude was significantly smaller at 13°C (*N* = 13, paired *t*-test, *p* < 0.001). (C) Left: Overlay of MCN1 ePSPs in LG at 10°C and 13°C. Right: Mean ePSP amplitudes at 10°C and 13°C for all preparations tested. Mean ePSP amplitude decreased significantly at 13°C (*N* = 13, paired *t*-test, *p* < 0.001). (D) Change in LG input resistance at 10°C and 13°C. (E) Membrane potential deflections of LG at 10°C and 13°C during de- and hyperpolarization (+3 to −3 nA, 1 nA steps are shown, 10 s duration). Measurements are from the same preparation. (F) Change in LG membrane potential as a function of current injection at 10°C and 13°C. Averages and standard error of the mean (SEM) of five experiments are shown. The starting membrane potential was set to −70 mV in all experiments. Note the difference in slope between 10°C and 13°C. Voltage deflections for all current levels were significantly smaller at 13°C (*N* = 5, paired *t*-test, *p* < 0.05).

The pyloric and gastric mill rhythms share the same main function: digestion of food. Proper digestion is vital for the animal’s survival, and it is intuitive to assume that both rhythms are equally important and that mechanisms exist to prevent cessation of both rhythms. For the pyloric rhythm, it has been suggested that physiological temperature compensation is achieved by opposing temperature dependencies of membrane currents (I_h_ and I_A_; [3]). The gastric mill rhythm, in contrast, apparently lacks adequate compensation despite the fact that pyloric and gastric mill neurons are located in the same ganglion and comprise comparable ion channels and membrane currents (e.g., I_h_ and I_A_ can be found in pyloric and gastric mill neurons; [26,27]). To determine what provoked the termination of gastric mill activity, we asked whether intrinsic factors contributed to the observed temperature-induced changes in LG activity. We first compared the intracellular response of LG to temperature changes: we found that LG’s resting potential hyperpolarized at 13°C (Fig 3A), with an average drop of 2.67 ± 1.34 mV (10°C: −67.01 ± 3.00 mV, 13°C: −69.68 ± 3.50 mV, *N* = 13). Hyperpolarization was continuous and linear with temperature increase (Fig 3A, right). Also, LG spike amplitude decreased significantly by 4.73 ± 1.22 mV (Fig 3B, 10°C: 17.02 ± 5.58 mV, 13°C: 12.28 ± 4.36 mV, *N* = 13). Next, we looked at the electrical postsynaptic potential (ePSP), which LG receives from MCN1 [12]. For this, MCN1 was stimulated with frequencies that did not elicit gastric mill rhythms, but rather only individual ePSPs (typically 1 Hz or below). Fig 3C shows an example of the change in ePSP amplitude when temperature was increased. On average, ePSP amplitude was reduced by 2.47 ± 0.54 mV at 13°C (10°C: 8.73 ± 3.06 mV, 13°C: 6.26 ± 2.52 mV, *N* = 13). All effects were reversible when temperature was decreased back to 10°C.

The MCN1 to LG gap junction has been shown to be voltage sensitive such that more hyperpolarized LG membrane potentials lead to smaller ePSPs. This could have possibly contributed to the diminishment of the ePSP amplitudes (since the resting potential hyperpolarized). However, this effect only leads to an average ePSP amplitude change of 0.14 mV/1 mV membrane potential change [12]. The temperature-induced change in ePSP amplitude in our experiments was almost six times larger (0.82 mV/1 mV). Thus, the observed change in LG membrane potential was not sufficient to explain the diminished ePSP amplitudes.

### Leak Conductance Increases at Higher Temperatures

The changes in resting potential, spike, and ePSP amplitude indicated that the input resistance of LG might have changed, causing a shunt of all of LG’s responses. We found that input resistance decreased significantly by 4.12 ± 1.4 MΩ (34.34 ± 10%, 10°C: 12.03 ± 2.38 MΩ, 13°C: 7.92 ± 2.12 MΩ, *N* = 13) when temperature was increased (Fig 3D). Changes in input resistance can be due to changes in leak currents, voltage-gated currents, or synaptic input. The latter appears unlikely to have contributed, since the STG was decentralized. Decentralization removes most spontaneous activity of descending projection neurons that may cause synaptic input to LG, and it stops the gastric mill rhythm and silences or strongly diminishes the pyloric rhythm. Consequently, because of the lack of STG activity in this condition, synaptic input from other STG neurons was also unlikely to have contributed to the observed change in input resistance. To further reduce STG and projection neuron input to LG, we blocked action potentials with tetrodotoxin (TTX) (0.1 μM; *N* = 4). The result was the same: we obtained a decrease in input resistance when temperature was increased. Hence, the decrease in input resistance was independent of synaptic input.

We also tested a broader range of current amplitudes by injecting 10 s long current pulses into LG, ranging from 1 to 3 nA in both the depolarizing and the hyperpolarizing direction. Fig 3E shows that the voltage deflections of all current steps were smaller at 13°C than at 10°C. This was true for all preparations tested (*N* = 5). Fig 3F shows the change in LG membrane potential as a function of the injected current. We noted a difference between LG’s voltage response to current injections in the positive and negative direction. While this has not been reported directly before, it is most likely a result of the aforementioned voltage dependence of the gap junction between LG and MCN1 [12]. Importantly, the resulting skew of LG’s voltage response in the tested current range was small in comparison to the shunting effect of temperature increase.

### Temperature-Dependent Changes in Leak Conductance Determine Network Oscillations

Since many, if not all, processes in the nervous system are temperature dependent, a causal connection between temperature effects on a specific process and the output of a motor circuit is difficult to show. Our data so far show that when temperature increases (1) leak conductance of LG increases, associated with (2) a hyperpolarization of LG’s resting potential. To test whether either of these two effects or both could contribute to the termination of the gastric mill rhythm at higher temperature, we first tested the effects of a change in membrane potential. For this, we recorded LG intracellularly and measured the membrane potential at 10°C and 13°C. We then elicited a gastric mill rhythm via MCN1 stimulation at 10°C and hyperpolarized LG to resting potential values obtained at 13°C (ΔV_m_ = 2.88 ± 1.55 mV, *N* = 4). The gastric mill rhythm was not affected by this manipulation. Thus, the observed change in membrane potential at 13°C was not a significant contributor to the termination of the gastric mill rhythm.

**Fig 4:**
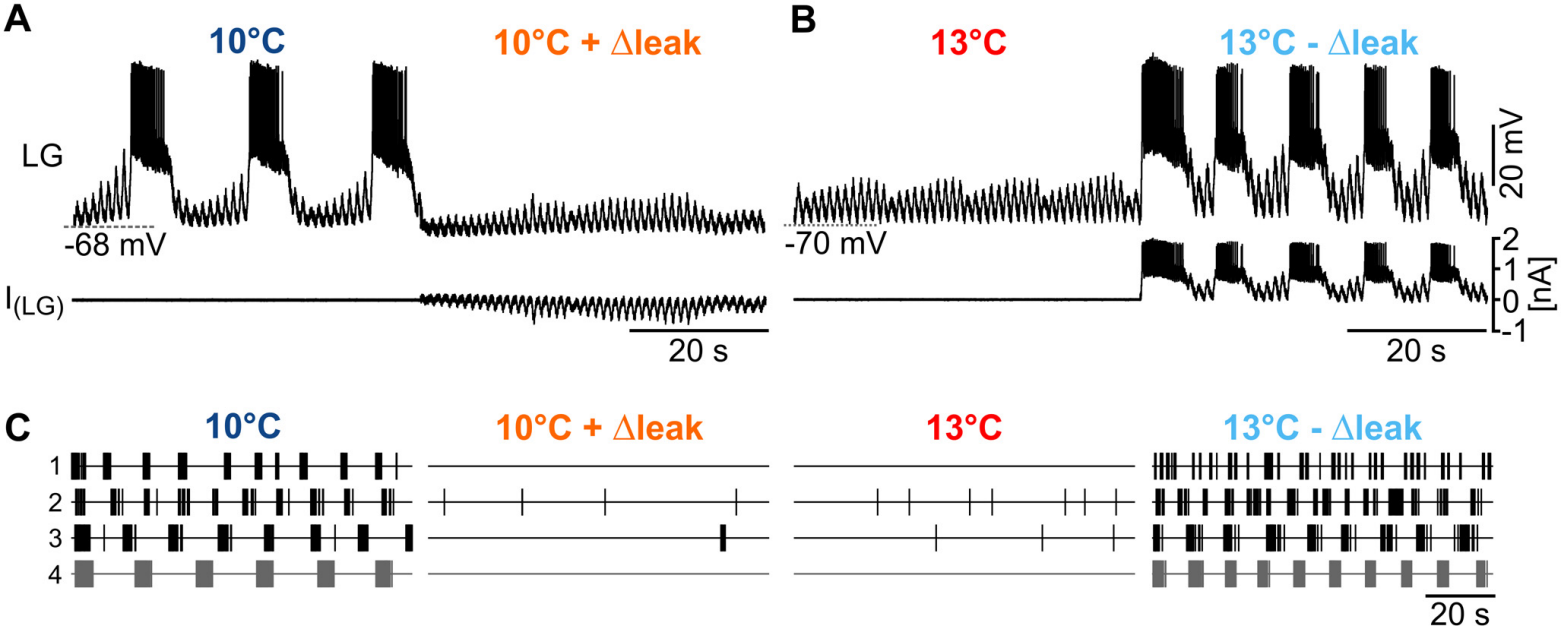
Changes in leak conductance are sufficient to terminate and rescue the rhythm. (A) Top: Intracellular recording of LG during tonic MCN1 stimulation with 7 Hz at 10°C. Rhythmic activity ceased when artificial leak was added with dynamic clamp (10°C + Δleak). Bottom: Corresponding dynamic clamp current that was injected into LG. (B) Top: Intracellular recording of LG during tonic MCN1 stimulation with 7 Hz at 13°C. Rhythmic activity was recovered when artificial leak was subtracted (13°C − Δleak). Bottom: Corresponding dynamic clamp current that was injected into LG. Traces in B and C are from the same preparation. (C) Effect of artificial leak addition (10°C + Δleak) and subtraction (13°C − Δleak) on LG spike activity for all tested preparations (1 to 4). Each vertical line represents an AP in LG over 100 s of continuous MCN1 stimulation. Grey traces (4) correspond to recordings shown in A and B.

We next tested whether an increase in leak conductance is sufficient to explain the termination of the gastric mill rhythm by using the dynamic clamp technique [28]. We either added an artificial leak conductance at 10°C or subtracted leak conductance at 13°C. First, we measured LG input resistance and resting potential at 10°C and 13°C and used the difference to calculate the leak conductance increase (= Δleak, see Materials and Methods). We then elicited a gastric mill rhythm at 10°C, and after several gastric mill cycles, we turned the dynamic clamp on and injected the appropriate amount of additional leak (+Δleak). Immediately after the onset of the artificial leak conductance, LG bursting ceased (Fig 4A). Thus, an increase in leak conductance as caused by a temperature increase of 3°C was sufficient to terminate the gastric mill rhythm.

Consequently, a reduction of leak conductance at high temperature should also be sufficient to restore the rhythm. We tested this prediction by carrying out the reverse experiment (Fig 4B). We stimulated MCN1 at 13°C with the threshold frequency that was sufficient to elicit a rhythm at 10°C. As seen in our previous experiments, no gastric mill rhythm was elicited at 13°C despite the continuous MCN1 stimulation. We then turned on the dynamic clamp and subtracted the appropriate leak (−Δleak). Immediately, LG regained its spiking ability, and rhythmicity was restored. In two out of the four experiments, LG firing stopped completely when artificial leak was added at 10°C (Fig 4C). In the other half, LG either generated sporadic action potentials or infrequent bursts of a few action potentials with varying interburst intervals. Thus, in all experiments MCN1 stimulation elicited a gastric mill rhythm at 10°C but failed to do so at 13°C (similar to our previous findings; see Fig 2). When leak was subtracted (13°C − Δleak), all preparations recovered the rhythm (Fig 4C, right). Consistent with the previous experiments (Fig 3), adding leak diminished action potential (AP) and ePSP amplitudes, while subtracting leak increased them.

In summary, our results demonstrate that a temperature-induced increase in leak conductance was sufficient to terminate the rhythm. Accordingly, bursting in LG could be restored at elevated temperatures by adding a negative leak.

### MCN1 Activity Increases with Elevated Temperatures

**Fig 5:**
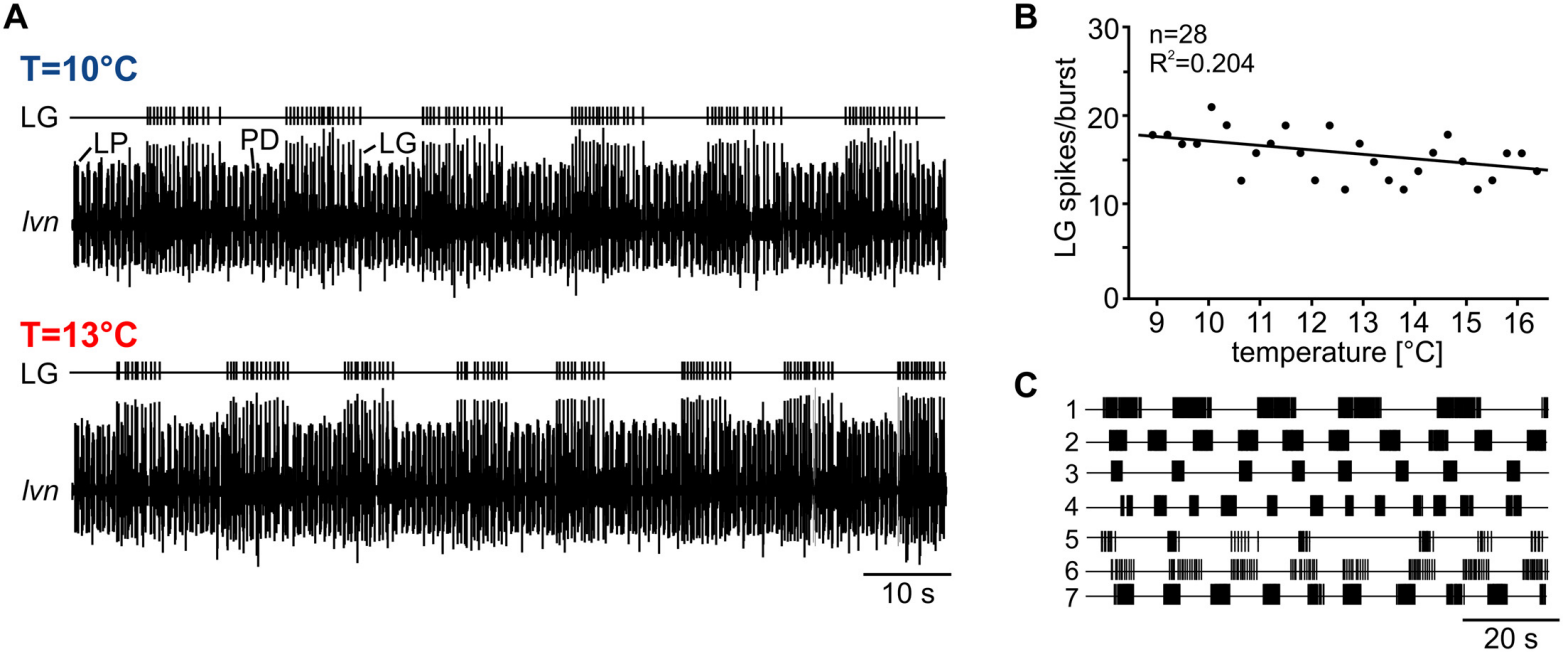
In vivo gastric mill rhythms occur at elevated temperature. (A) Extracellular recording of the *lvn* in an intact animal with spontaneous pyloric and gastric mill activity at 10°C (top) and 13°C (bottom) during fast temperature change (~1°C/min). As access to the motor nerves is limited in vivo, LG activity was assessed on the *lvn*. LG spikes are superimposed on those of the pyloric neurons (LP and PD are clearly discernable). For visualization, LG spikes are shown as vertical lines above the recording trace. (B) LG spikes/burst in vivo as a function of temperature from 9°C to 16°C during fast temperature increase (~1°C/min). Regression slope: −0.46*LG spikes/burst, slope significantly different from 0 with *p* < 0.049. (C) LG spike activity in vivo at 13°C for all animals tested (*N* = 7). Here, temperature was increased slowly (1°C/h) from 10°C to 13°C. Each trace represents one animal, and each vertical line an LG action potential.

*C*. *borealis*, the animal used for this study, experiences substantial temperature fluctuations in its habitat [29,30]. One would thus assume that the nervous system should be able to cope with the small temperature fluctuations we applied in our experiments. To test if there may be mechanisms to compensate for the temperature-induced termination of the gastric mill rhythm in vivo, we implanted extracellular electrodes in intact animals and recorded the main motor nerve (*lvn*). Recordings typically lasted for several days. We investigated temperature effects on the gastric mill rhythm using two approaches: first, the temperature of the water was changed at a rate comparable to the in vitro experiments (1°C/min)—a velocity that has previously been shown to be sufficiently slow to cause similar changes at the STG somata [18]. Since we were interested in spontaneous gastric mill rhythms, i.e., rhythms that were independent of artificial stimulation, temperature was only increased after a gastric mill rhythm was present at 10°C. The rhythm was then continuously monitored during the temperature change. Fig 5A shows the rhythm obtained at 10°C and 13°C. In contrast to the in vitro condition, the rhythm persisted in vivo and showed no signs of irregularity. The number of LG spikes per burst declined slightly with increasing temperature (Fig 5B), but the number of LG bursts increased at the same time, which was in stark contrast to the isolated nervous system.

Yet, physiological temperature changes might occur over longer periods. Thus, in a second set of experiments, the temperature was kept at 10°C for at least 1 h and then slowly increased at a rate of 1°C/h to 13°C (similar to Fig 2D). We found that in these conditions spontaneous gastric mill rhythms occurred at 13°C (*N* = 7, Fig 5C), implying the existence of mechanisms that compensate the temperature-induced changes in the gastric mill circuit in vivo.

**Fig 6:**
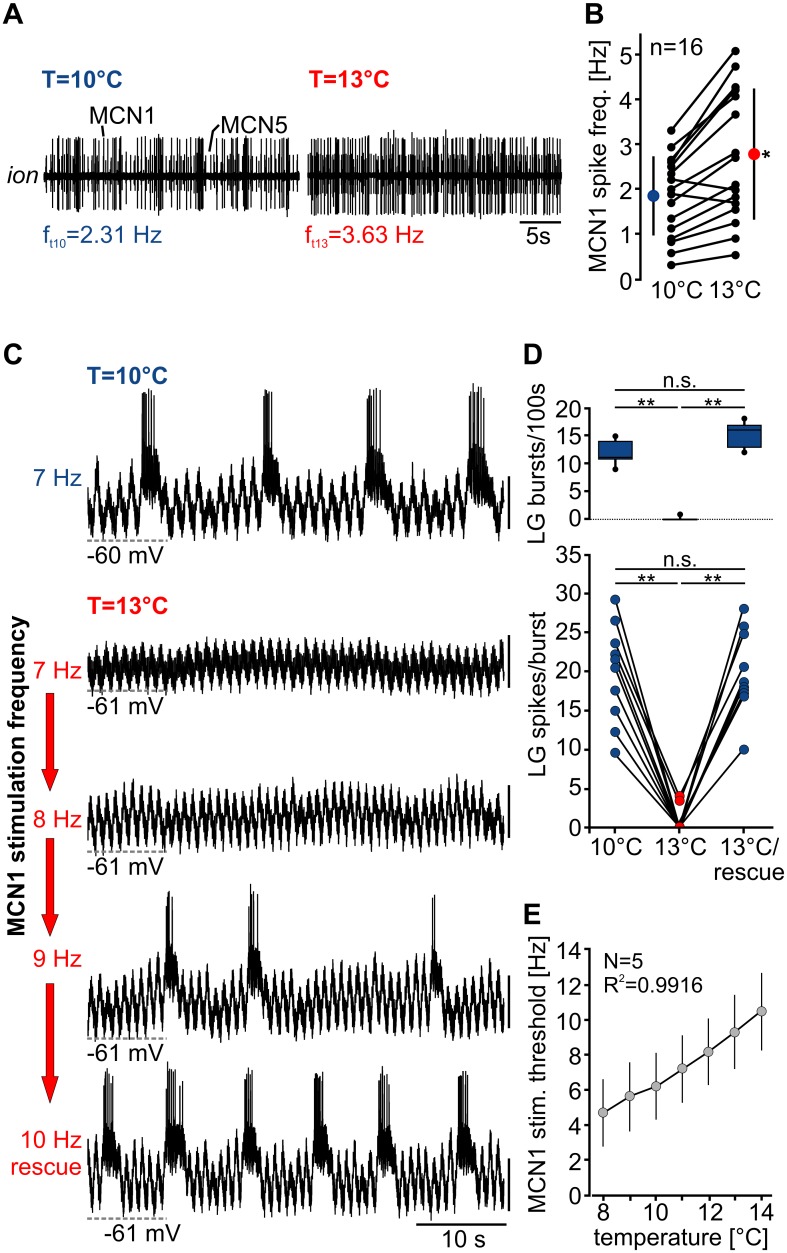
Increased MCN1 activity rescues the rhythm from temperature-induced breakdown. (A) Extracellular recording of the *ion* showing the spike frequency *(f)* of MCN1 at 10°C and 13°C. CoGs were isolated from the STG by transecting all connecting nerves. MCN1 activity was recorded on the remaining stump of the *ion* that was still connected to the CoG. (B) Change in MCN1 spike frequency at 10°C and 13°C for *N* = 8 preparations. In each preparation, both *ions* were recorded (*n* = 16 recordings of bilateral MCN1). Black circles represent individual experiments; colored circles are means ± SD. MCN1 frequency was significantly higher at 13°C (*N* = 8, *n* = 16, paired *t*-test, *p* < 0.05). (C) Intracellular recording of LG at 10°C (top trace) and during increasing MCN1 stimulation frequency at 13°C (subsequent traces). Traces are from the same preparation. Vertical scale bars, 10 mV. Rhythmic LG activity was lost at 13°C but could be recovered by increasing MCN1 stimulation frequency. (D) Number of LG bursts/100 s (top) and LG spikes/burst (bottom) of all preparations at 10°C, 13°C, and 13°C with increased MCN1 stimulation frequency (= 13°C/rescue). Values at 10°C and 13°C/rescue were significantly different from 13°C (*N* = 10, Friedman RM ANOVA on ranks, χ^2(2)^ = 15, *p* < 0.001, Tukey post hoc test with *p* < 0.01 overall significance level (top) and one-way RM ANOVA, F(2,18) = 79.199, *p* < 0.001, Holm-Sidak post hoc test with *p* < 0.01 (bottom). (E) Change in minimum MCN1 stimulation frequency needed to induce rhythmic LG activity (= “stim. threshold”) as a function of temperature. Means ± SD are shown (*N* = 5). Regression slope: 0.96*stim threshold, slope significantly different from 0 with *p* < 0.001.

To mechanistically understand the adaptations rescuing the gastric mill rhythm, we went back to the in vitro preparation. Since there was no apparent compensation within the STG circuit, we focused on one of LG’s modulatory input, namely MCN1. MCN1 had been the only projection neuron providing input to LG in our experimental setup (see also [11]), and our initial experiments had already indicated that increasing MCN1 stimulation frequency increased the dynamic range of the gastric mill rhythm (Fig 2E). To scrutinize this idea and to test if a temperature-dependent up-regulation of MCN1 projection neuron activity could counterbalance the termination of the gastric mill rhythm, we first determined MCN1 activity at different temperatures. We recorded spontaneous MCN1 spike activity in preparations in which feedback from the pyloric and gastric mill CPGs in the STG was severed to exclude ascending influences on the activity of MCN1 [31]. In contrast to the previous experiments, we now altered the temperature of the CoG. MCN1 activity was recorded extracellularly from the *ion* stump connected to the CoG. We found that MCN1 activity increased at 13°C. In the example in Fig 6A, MCN1 firing frequency increased by 57.59%. Note that the activity of both MCN1 neurons in a given nervous system preparation was analyzed to determine if temperature affects both MCN1 copies similarly. Although MCN1 firing frequency at 10°C was quite variable between the two MCN1 neurons within a given preparation and across animals, in seven of eight preparations, firing frequency of both MCN1 neurons increased at 13°C (by 51.29 ± 26.59%, *N* = 8, *n* = 16, Fig 6B).

### Temperature-Induced Increase in MCN1 Activity Rescues Rhythms

Next, we asked whether the temperature-induced up-regulation in MCN1 firing frequency is sufficient to counterbalance the increase in LG leak conductance and to prevent the termination of the gastric mill rhythm at elevated temperatures. To test this, we went back to the original experimental setup in which we decentralized the STG from all CoG inputs and stimulated the *ion* on the STG side of the nerve transection to elicit gastric mill rhythms (Fig 1A). Specifically, we stimulated MCN1 at 10°C with threshold frequency, observed the rhythm, and monitored its cessation after increasing the STG temperature to 13°C. We then raised the MCN1 stimulation frequency in 1 Hz steps to mimic the increase in MCN1 firing frequency observed at 13°C. Fig 6C shows that an increase in MCN1 firing frequency from 7 to 10 Hz (42.85%) was sufficient to restore the rhythm in this particular example. On average, a 56.07 ± 11.99% (*N* = 10) increase in MCN1 stimulation frequency rescued the gastric mill rhythm. This was true although threshold MCN1 stimulation frequencies varied considerably between preparations (5–9 Hz at 10°C, 8–15 Hz rescue frequency at 13°C). We found a significant decrease in the number of LG bursts and LG spikes per burst at 13°C, but both returned to control values when MCN1 stimulation frequency was increased (Fig 6D). In fact, when we measured the minimum MCN stimulation frequency at different temperatures (Fig 6E), we found that a linear increase in MCN1 frequency of 0.96 Hz/1°C was sufficient to rescue the rhythm at increasing temperatures.

### Neuromodulator Application Can Rescue the Gastric Mill Rhythm

**Fig 7:**
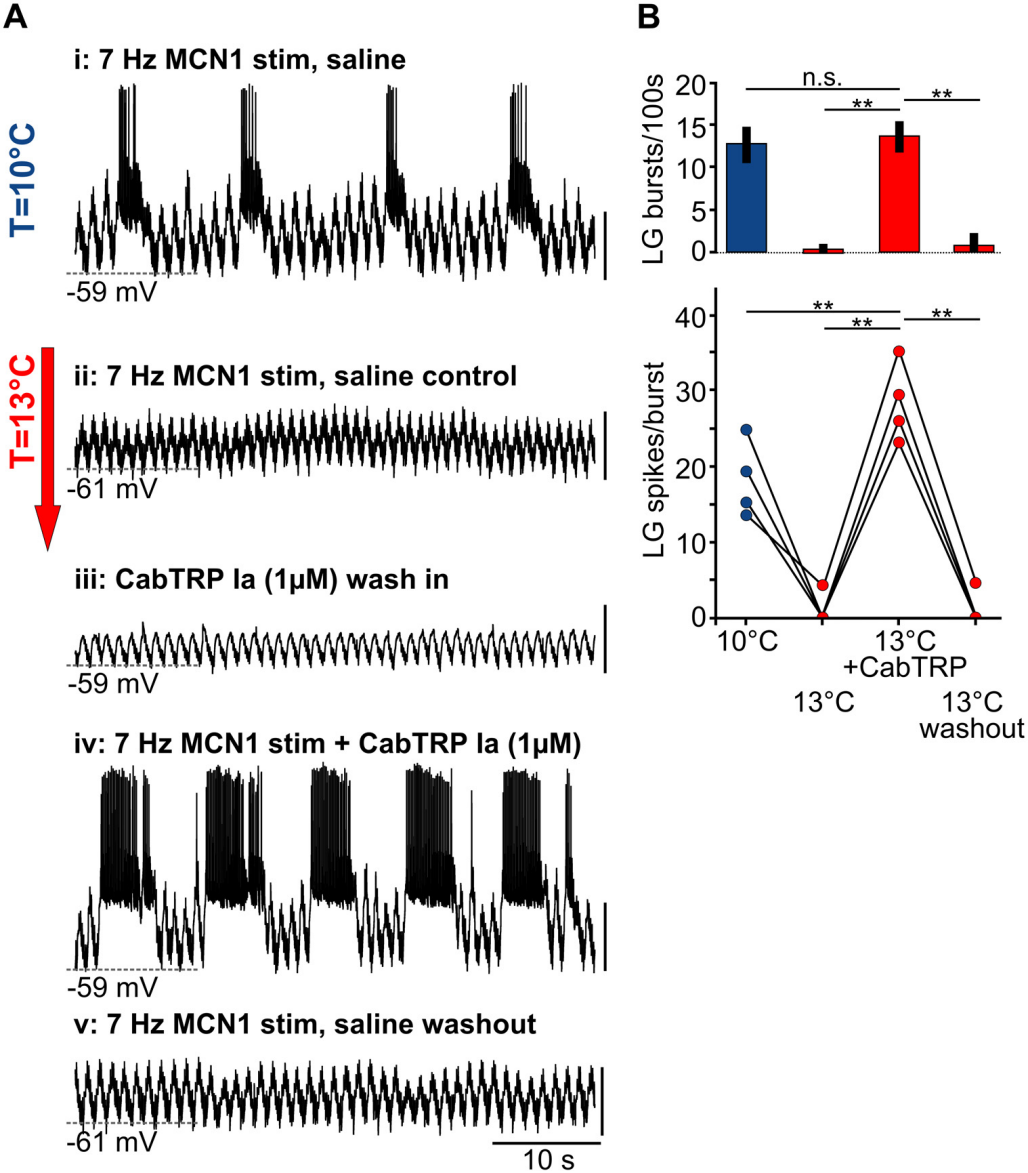
CabTRP Ia application rescues the rhythm from temperature-induced breakdown. (A) Intracellular recording of LG at 10°C (top trace) and 13°C (subsequent traces) and response to 1 μm CabTRP Ia at 13°C in the absence and presence of tonic MCN1 stimulation (7 Hz). All traces are from the same preparation. Rhythmic LG activity was lost at 13°C but could be recovered in CabTRP Ia without increasing MCN1 stimulation frequency (iv). Vertical scale bars, 10 mV. (B) Change in number of LG bursts/100 s (top) and LG spikes/bursts (bottom) at 10°C, 13°C, 13°C + CabTRP Ia and after washout. *N* = 4, One Way RM ANOVA, F(3,9) = 50.558 (top) and F(3,9) = 124.33 (bottom), *p* < 0.001, Holm-Sidak post hoc test with *p* < 0.01 significance level.

What is the mechanism that allows MCN1 to rescue the rhythm at 13°C? Our previous results indicate that subtracting a leak conductance is sufficient to achieve this goal (Fig 4B). Bursting in LG is mainly driven by the release of MCN1’s peptide cotransmitter CabTRP Ia [11]. In the STG, CabTRP Ia is exclusively found in the MCN1 terminals and thus is specific to MCN1. Like many other modulators in the STG, CabTRP Ia activates a well-characterized voltage-gated cation conductance (I_MI_, modulator-induced current; [17]). I_MI_ supports membrane potential oscillations because of its inverted bell-shaped voltage-current relationship [32]. Importantly, I_MI_ has recently been suggested to act as a negative leak conductance because of the linear falling edge of its voltage-current relationship [33]. Could the CabTRP Ia-activated I_MI_ be sufficient to rescue the rhythm by counterbalancing the temperature-induced leak increase in LG? To test this, we bath applied CabTRP Ia (1 μM; [11]) as a means to increase I_MI_ and measured the response of LG during MCN1 stimulation at 13°C. The release concentration and dynamics of CabTRP Ia are unknown, and the effective concentrations of peptide transmitters on STG neurons differ greatly between neuron types [34]. As current responses to peptide modulators also vary substantially from animal to animal [35], we made no attempt to determine the CabTRP Ia threshold concentration. Rather, and most importantly for our purposes, we used a concentration shown to be effective in activating I_MI_ [15]. We first elicited a rhythm at 10°C, then increased the temperature to 13°C to elicit the termination, and finally applied CabTRP Ia. Fig 7A shows that CabTRP Ia application indeed can restore the rhythm. In the example shown, 7 Hz MCN1 stimulation elicited a gastric mill rhythm at 10°C (Fig 7A, i), but not at 13°C (ii). We then stopped the MCN1 stimulation and applied CabTRP Ia. CabTRP Ia alone never elicited a gastric mill rhythm nor did it cause LG action potentials (iii). CabTRP acts specifically on I_MI_, a G-protein coupled voltage-dependent inward current [14,34]. Hence, to cause sustained LG activity, an additional depolarization of the membrane potential would be required. We noted a consistent small depolarization of the membrane potential (2.01 ± 0.97 mV, *N* = 8), which is consistent with earlier findings [15] and neuronal release of CabTRP Ia by MCN1 [11], indicating that the concentration used was within the physiological range used by MCN1. We also observed subthreshold oscillations in the LG membrane potential as a result of rhythmic disinhibitions from Int1 that were triggered by increased pyloric activity in the presence of CabTRP Ia [17]. When MCN1 stimulation was turned on (iv), however, LG responded immediately to the threshold stimulation frequency and generated rhythmic bursts of action potentials at 13°C. The effects of CabTRP Ia on LG were reversible (v), i.e., MCN1 stimulation at 13°C with the threshold frequency after CabTRP Ia washout was neither sufficient to elicit LG spikes nor to start a gastric mill rhythm. Across animals (*N* = 4), we found that CabTRP Ia application always restored rhythmic LG activity at 13°C when MCN1 was activated with the threshold frequency. Correspondingly, the number of LG bursts and the number of LG spikes per burst first decreased significantly at 13°C (Fig 7B) and then increased in the presence of CabTRP Ia. We noted similar but weaker effects in experiments with lower CabTRP Ia concentrations (N = 4). In two preparations, rhythmic LG activity recovered at 100 nM, and LG firing frequency was not significantly different from the 10°C control. Lower concentrations did not elicit spiking in LG at elevated temperatures in those two experiments. In the other two experiments, the rhythm recovered at 10 nM, but the firing frequency of LG was lower when compared to the 10°C control condition, indicating that 10 nM did not fully recover LG rhythmicity at elevated temperatures. Only with a simultaneous increase in MCN1 stimulation frequency were control values reached.

MCN1 activation in all experiments was necessary to elicit the rhythm, independently of whether CabTRP Ia was present or not. Thus, MCN1's additional transmitter release and network effects (such as activating the LG half-center antagonist Int1) were necessary to start the rhythm. In summary, the CabTRP Ia-induced I_MI_ broadened the permissible temperature range of the gastric mill rhythm and allowed LG to generate rhythmic bursts of activity.

### The Balance of I_MI_ and Leak Currents Determine Network Oscillations

Our results show that CabTRP Ia is sufficient to counterbalance the temperature-induced leak current in LG and to rescue the rhythm at 13°C, presumably by its known effect on I_MI_. We tested the dynamics and range of this compensation by using computational models of the gastric mill network with the known connectivity of the circuit (Fig 1A; [36]).

**Fig 8:**
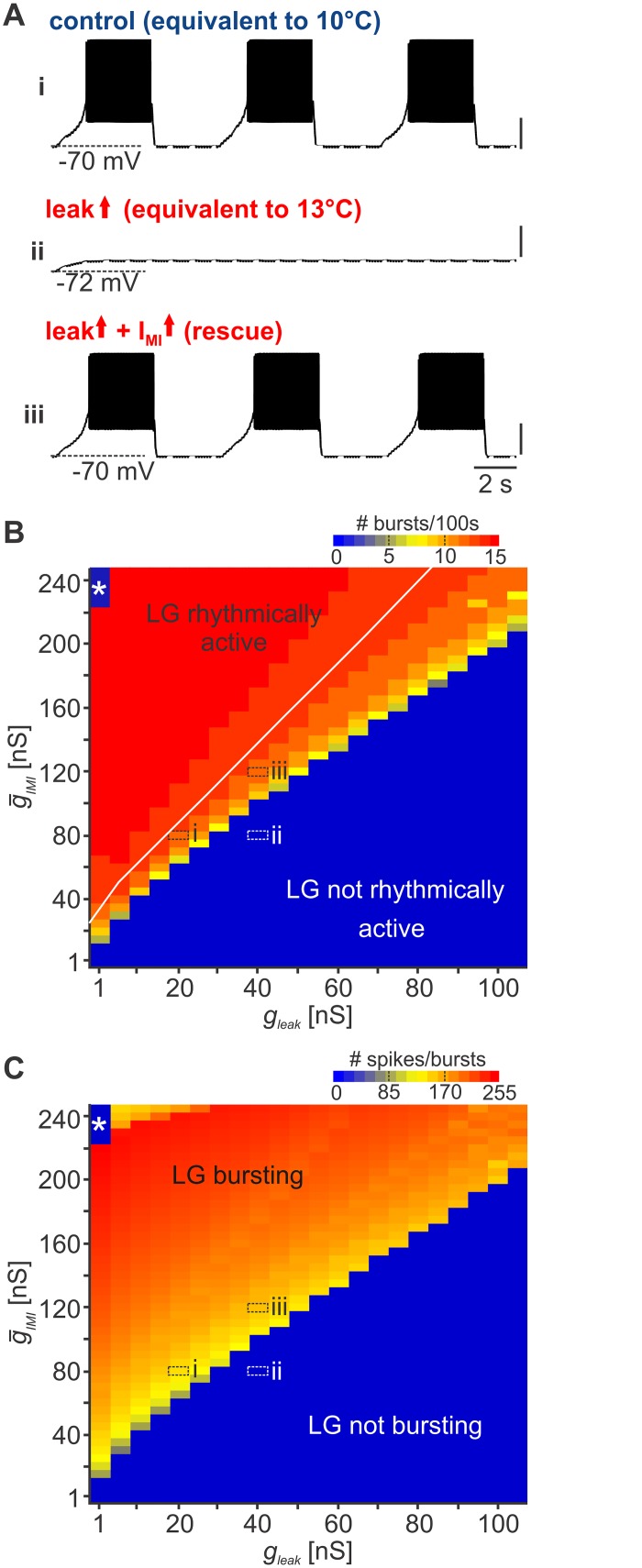
I_MI_ counterbalances leak conductance to rescue neural oscillations in a computational model. (A) Model output of LG membrane potential at (i) low leak and I_MI_ conductance (*g*_*leak*_ = 20 nS, ${\bar{g}}_{IMI}=80\text{ ns}$), (ii) increased leak (*g*_*leak*_ = 40 nS, ${\bar{g}}_{IMI}=80\text{ ns}$), and (iii) increased I_MI_ (*g*_*leak*_ = 40 nS, ${\bar{g}}_{IMI}=120\text{ ns}$). Vertical scale bars, 20 mV. (B) Color map of number of bursts occurring in 100 s for 1,100 simulations as a function of leak and I_MI_ conductances. Warmer colors represent more bursts;* highlights areas with one burst. (C) Color map of the number of LG spikes/100 s for 1,100 simulations as a function of leak and I_MI_ conductances. Warmer colors represent more LG spikes. (B–C) Labeled areas (i–iii) indicate parameter sets corresponding to the traces shown in A.

Pyloric influences on the gastric mill network were modeled by driving the pyloric pacemaker neuron AB with constant sinusoidal currents. Initially, using values within the physiological range, we set leak and I_MI_ conductances such that the model produced oscillations that were similar to the biological network (Fig 8A, i). To mimic the effects of a temperature increase, we then added an additional leak conductance to the model LG. With twice the amount of leak, rhythmicity was absent, and LG was completely silent (ii). All other parameters were intentionally kept constant. No temperature-dependent changes other than the increase in the leak current (as indicated by Fig 3F and Fig 4) were added since the goal was to test the interplay between leak and I_MI_ conductances rather than the effect of temperature on synaptic and membrane properties other than leak. When we increased the I_MI_ maximum conductance by 50%, rhythmicity was restored (iii), indicating that I_MI_ indeed can counterbalance the leak.

To determine whether this was true for a greater range of parameter combinations and to identify the borders of stable network oscillations, we carried out an exhaustive search of 1,100 LG models. Model neurons varied in terms of the maximal conductances of leak and I_MI_ currents but were otherwise identical. This allowed us to test the effects of various combinations of leak and I_MI_ conductances on the behavior of the network. Leak and I_MI_ conductance levels were independently varied in steps of 5 nS, starting at 1 nS. To scrutinize model activity, we analyzed rhythmicity and the number of LG spikes and bursts in 100 s simulation time. Models were classified as rhythmic when at least three clearly identifiable bursts were present within 100 s. Bursts were defined as series of action potentials followed by interburst intervals of more than 2 s. We plotted the number of bursts for all 1,100 models as a function of leak and I_MI_ conductances (Fig 8B). In blue areas, the model LG neuron was either not spiking or not rhythmically active (area highlighted with *), whereas in yellow and red areas the model output was rhythmically active. For each leak conductance tested, an increase in I_MI_ was able to restore rhythmicity. Increasing leak conductance required larger I_MI_. Transitions in model activities were abrupt, i.e., within small changes in conductance levels, models either produced regular bursting or they were silent. This bimodal behavior most likely reflected the properties of the network that elicited oscillations in the first place. Increasing I_MI_ beyond the minimum level for rhythmicity elicited more bursts and sped up the rhythm. The number of LG spikes increased with higher I_MI_ levels and decreased with higher leak (Fig 8C), indicating that the parameter combination of leak and I_MI_ determined not only the rhythmicity of the model but also burst and spike frequency. Only very few models (five out of 1,100) with high I_MI_ and low leak produced nonrhythmic activities with a single, high frequency burst (* in Fig 8B and 8C). Here, the spike number within the one burst dropped with higher I_MI_, indicating that action potentials could no longer be generated because of sodium channel inactivation.

The model also indicates how closely tuned conductances in the biological system are. In our experiments, we saw a 52% average increase in LG leak conductance (= 34% decrease in input resistance, see Fig 3D) at 13°C. This increase was sufficient to stop the rhythm. At 10°C, I_MI_ must have been sufficiently high to elicit oscillations but low enough that a 52% increase in leak conductance was sufficient to terminate them. This fact allowed us to estimate the maximum I_MI_ conductance for each leak value to identify parameter combinations sufficient to explain the temperature-induced termination observed in the biological system. Although higher I_MI_ values would allow oscillations (with faster rhythms and more action potentials), they would not lead to a termination of the rhythm with the average amount of leak increase that was observed during temperature elevation. The white line in Fig 8B depicts the maximum I_MI_ for all tested leak conductances. Maximum I_MI_ was always close to the threshold for oscillations, indicating that leak and I_MI_ may be closely balanced in the biological system. The analysis of the model also gives an interpretation of the CabTRP Ia experiments: bath application of the modulator led to faster and stronger bursting than in the 10°C control situation (Fig 7), indicating that I_MI_ increased beyond control values (a vertical shift from yellow to red areas in Fig 8B).

The almost linear progression of the threshold between bursting models and silent ones (transition between blue and yellow in Fig 8B) indicates that I_MI_ indeed acted mostly in a linear fashion to counterbalance the leak. This suggests that the two conductances may be coregulated when temperature changes in the biological system to enable stable motor patterns.

In conclusion, our analysis shows that increases in leak conductance can be counterbalanced by higher I_MI_ to restore network oscillations. This effect, although independent of temperature by itself, seems to be a crucial mechanism to compensate temperature-induced leak conductance changes for the rescue of the gastric mill rhythm. I_MI_ has been suggested to support membrane potential oscillations by acting as a negative leak conductance [33]. It appears that this property can facilitate compensation of the temperature-induced leak increase in LG.

In summary, I_MI_ acts as a negative leak to counterbalance changes in leak conductance and to facilitate rhythmicity in neuronal oscillators. In the case of the gastric mill rhythm, I_MI_ is most likely increased by the actions of CabTRP Ia released from MCN1 during higher temperature-induced neuronal activity. This rescues oscillations in the gastric mill CPG and might be responsible for the broader temperature tolerance in vivo.

## Discussion

All animals experience temperature challenges and need mechanisms that prevent detrimental effects on nervous system function. Since all cellular processes including synaptic and intrinsic membrane currents are influenced by temperature, the nervous system faces the challenge of maintaining function when temperature changes. The phase relationship of the pyloric CPG is maintained over a wide temperature range irrespective of large cycle period changes, and it has been proposed that well-regulated temperature effects on opposing intrinsic factors facilitate this phase maintenance [3]. However, in those experiments, temperature also affected extrinsic modulatory inputs, which may contribute to the observed phase constancy. When we specifically altered temperature of the CPG network and kept extrinsic neuromodulatory inputs unaffected, the pyloric network showed the same phase constancy (S1 Fig) as previously described. Balancing opposing intrinsic factors thus seems to be sufficient to account for the robustness of the pyloric pattern in the temperature range tested here. However, it is possible that temperature-dependent changes in neuromodulatory input alter, shift, or extend the permissive temperature range of the network with the result that phase constancy is maintained over a wider temperature range.

In contrast, the permissive temperature range of the gastric mill network was small (less than 3°C, Fig 2). However, gastric mill activity persisted at elevated temperatures when extrinsic input was adjusted (Fig 6). Hence, temperature compensation in this circuit is highly dependent on extrinsic input. The main effect of the extrinsic input appears to be a compensation of a temperature-induced decrease in input resistance. Temperature effects on input resistance have been described in other systems: input resistance in goldfish Mauthner cells, for example, is consistently smaller in animals when acclimated to warm temperatures [37]. In LG, the changes in membrane potential in response to de- and hyperpolarizing current injections reveal that voltage responses were shunted over a broad range of membrane potentials (Fig 3F). Recent studies have shown that changes in leak currents play an important role for network oscillations [32,38–40], regulation of excitability [41,42], and switches in the activity states of neurons [43]. Our dynamic clamp results support the idea that the regulation of leak currents determines the ability of LG to generate oscillations: first, an increase in leak conductance was sufficient to terminate oscillations in LG (Fig 4A) and in the model (Fig 8). Second, when a leak current with appropriate negative conductance was injected in LG, the rhythmic pattern of the entire gastric network was recovered (Fig 4B). The modulation of leak currents by neurotransmitters has been proposed to contribute to the regulation of neuronal excitability [44–46]. Nevertheless, few studies have directly tested the dependence of network oscillations on leak currents and the role of neuromodulators in this process. This study for the first time provides direct evidence that extrinsic neuromodulation counterbalances leak currents to restore network oscillations. Activating the neuromodulatory projection neuron MCN1 with higher frequencies could restore rhythmic activity at elevated temperatures (Fig 6). The frequency increase needed to restore rhythmic activity was in the same range as temperature increases spontaneous MCN1 activity (~50%; Fig 6A). MCN1 excites LG via peptidergic (CabTRP Ia) activation of I_MI_ [11,47]. CabTRP Ia application was sufficient to restore network oscillations at higher temperatures (Fig 7), showing that neuromodulator release can adjust the permissive temperature range of the system to facilitate robustness.

I_MI_ is a second messenger activated voltage-dependent inward current similar to that evoked by N-Methyl-D-aspartate (NMDA) [14]. It is blocked at hyperpolarized membrane potentials by extracellular Ca^2+^ and has a reversal potential of about 0 mV, resulting in an inverted bell-shaped current-voltage relationship. The negative slope in a portion of its current-voltage relationship has recently been shown to act as a negative leak conductance [32] and to restore oscillations when artificially injected into pyloric network pacemaker cells. Our models show that an increase in I_MI_ was sufficient to restore the leak-induced termination of rhythmic activity (Fig 8A) that was observed during a temperature increase. This was true for a great range of parameter combinations (Fig 8B). I_MI_ itself will likely increase with higher temperature (as most other currents), but even if this was the case in our experiments, this increase was not sufficient to counterbalance the temperature-induced leak increase in LG. Rather, the rhythm could only be restored by (further) increasing I_MI_ via additional CabTRP Ia, either released from MCN1 when stimulated with higher frequencies or applied to the bath. The almost linear course of the threshold between bursting and silent models implies that I_MI_ indeed acted mostly in a linear fashion. Thus, the regulation of I_MI_ and leak seems to be well adjusted by a balanced regulation of neuromodulator activity and cell-intrinsic conductances in the biological system to enable rhythmicity when temperature changes.

CabTRP Ia application and MCN1 stimulation also activate I_MI_ in pyloric neurons [14]. In preparations with intact extrinsic input, leak conductance increases in these neurons when temperature increases [3]. It is thus conceivable that I_MI_ also supports rhythmicity in the pyloric network by counterbalancing leak [32] and contributes to the phase constancy and robustness of the pyloric pattern. Yet, our data from decentralized preparations indicate that for the temperature range tested, I_MI_ actions are not necessary to maintain phase constancy (S1C Fig). Our models suggest, however, that if I_MI_ is present (i.e., when extrinsic inputs are available), robustness against leak-based perturbations increases. Potentially, thus, I_MI_ could act to shift or extend the permissive temperature range of the pyloric system in preparations with intact extrinsic input.

Temperature-induced effects on synaptic dynamics and voltage-gated conductances have been discussed previously and can contribute to temperature compensation and, in particular, facilitate phase constancy in rhythmic networks [3,4]. In the gastric mill rhythm, the dynamics of the synaptic connectivity mostly determine phasing and speed of the rhythm [48]. Temperature changes will naturally affect synaptic dynamics and voltage-gated ionic conductances. However, since a linear leak injected with dynamic clamp at 10°C was sufficient to stop the rhythm (Fig 4A), effects on synapses and other conductances could not have contributed significantly to the termination of the rhythm. Similarly, at 13°C, subtraction of a linear conductance was sufficient to restore the rhythm (Fig 4B). However, the speed of the rhythm and burst durations were indeed different from controls (Figs 6 and 7), indicating that temperature-dependent effects on intrinsic or network properties other than the leak contributed to the expression of the gastric mill rhythm. Yet, other conductances were not required for temperature compensation of rhythmicity.

### Extrinsic Stabilization of Network Activity

When body temperature varies, neuronal compensatory mechanisms are crucial to maintain nervous system function. Even in homeotherms, body temperature fluctuates in a daily and monthly fashion, albeit within a small range. This small range of experienced temperature may lead to a small permissible range in which temperature effects can be compensated. This is especially interesting for circuits that drive vital body functions: hyperthermia associated with pathological conditions such as fever and heat stroke can, for example, cause dysfunction of the breathing CPG and induce apnea [49]. In infants, in whom 85% of the body heat loss is accommodated by the head, apnea and sudden infant death syndrome occur more frequently when the head is heavily wrapped during sleep [50]. In fact, pacemaker neurons in the respiratory neural network are temperature sensitive [49], but only when synaptically isolated, suggesting that synaptic influences may help to counterbalance temperature effects. As with most rhythmic motor systems, the respiratory network is synaptically innervated by descending modulatory pathways [51].

Neuromodulation modifies network and synaptic properties on various time scales and has been shown to be involved in motor pattern selection and sensory functions that underlie behavioral performance. Often, the global presence or absence of a neuromodulator is equivalent to a specific behavioral state. Our study is the first to show that descending neuromodulation can also be crucial in temperature compensation of an oscillatory neural network. Descending modulation activated a cellular property of opposite sign to the temperature-induced intrinsic changes, making the compensation conditional on the activity of extrinsic input. The pyloric rhythm appears to be intrinsically compensated for larger temperature ranges because of network properties whose response properties change similarly with temperature but have opposing effects. Such mechanisms may be optimal for networks that are required to continuously produce rhythmic activity. For episodic pattern generators such as the gastric mill circuit, temperature compensation may rather be achieved by using the already present descending extrinsic innervation of the pattern generators that exert sophisticated modulatory control over the generated activity. Thus, two quite distinct mechanisms, one depending on the characteristics of the individual components of the network and the other emerging from the effects of descending modulatory fibers, can either individually or in combination compensate for temperature changes to maintain the output of a physiological system. Involving the neuromodulatory system may also allow more flexibility in response to temperature challenges: most descending neuromodulatory pathways integrate information from multiple sensory modalities, rendering temperature compensation conditional on sensory and behavioral conditions. This provides the opportunity to adjust type and strength of the compensation, much more so than with the inherent and rather inflexible compensation provided by intrinsic characteristics of the network.

## Materials and Methods

### Animals

Adult *C*. *borealis* were purchased from Ocean Resources (Sedgwick, Maine) or Fresh Lobster Company (Boston, Massachusetts) and maintained in filtered, aerated artificial seawater at 11°C before use.

### Dissection

Animals were anesthetized on ice for 20–40 min. For in vitro experiments, the stomatogastric nervous system was isolated from the animal according to [52], pinned out in a silicone-lined (Wacker) petri dish, and continuously superfused with physiological saline (11°C). We worked with fully intact and decentralized STNS preparations. In the latter, the STG was separated from the CoGs by transecting the paired *ion* and *son*.

For in vivo electrode implantation, anesthetized crabs were immobilized in a custom-built holder. Surgery was performed according to previously published protocols [18]. In short, animals were surrounded by ice to maintain anesthesia during surgery. A 3 x 3 cm window was cut into the dorsal carapace to expose the lateral ventricular nerve (*lvn*). A hook electrode was placed around the *lvn*, and the surgery site was sealed with Parafilm. For recovery, animals were placed back into the tank for at least 1 day. Neuronal activity was continuously recorded for several days in unrestrained animals.

*C*. *borealis*, the animal used in this study, is not subject to ethics approval at Illinois State University. While *C*. *borealis* is not a protected species, we still adhered to general animal welfare considerations regarding humane care and use of animals while conducting our research. Crabs were delivered from Massachusetts or Maine via UPS Express Over Night Shipping. For transportation, the animals were covered with wet sea grass and cooled on ice as appropriate for the species. After arriving, the crabs were housed for a maximum of 16 d in 12 tanks (each with a holding capacity of 100 gallons) at 10°C to 12°C as appropriate for the species. Water quality, salinity, and temperature were monitored daily. We never kept more than six animals in one tank. The crabs were then euthanized using ice, which is a method recognized as acceptable under the AVMA guidelines for euthanasia of aquatic invertebrates. All animals were confirmed dead before use.

### Solutions

*C*. *borealis* saline was composed of (in mM) 440 NaCl, 26 MgCl_2_, 13 CaCl_2_, 11 KCl, 10 Trisma base, and 5 maleic acid, pH 7.4–7.6 (Sigma Aldrich). In some experiments, 0.1 μm TTX (Alomone Labs) or 10 nM to 1 μM CabTRP Ia (GenScript) was added to the saline. Solutions were prepared from concentrated stock solutions immediately before the experiment. Stock solutions were stored at −20°C in small quantities. Measurements were taken after 45 min wash in/out.

### Electrophysiology

In vitro recordings were performed using standard methods [53–55]. Extracellular signals were recorded, filtered, and amplified with an AM Systems amplifier (Model 1700). Intracellular recordings were obtained from STG cell bodies using 10–30 MΩ glass microelectrodes (Sutter 1000 puller, 0.6 M K_2_SO_4_ + 20 mM KCl solution) and an Axoclamp 900A amplifier (Molecular Devices) in bridge or two electrode current clamp mode. Files were recorded, saved, and analyzed using Spike2 Software at 10 kHz (version 7.11; CED). Input resistance was measured using hyperpolarizing current pulses (1 nA, 500 ms duration). Membrane potential voltage deflections were measured in steady state (after 500 ms). In some experiments, current injections ranging from −3 nA to +3 nA in 0.5 nA steps were used. In this case, current pulses were 10 s long, and interpulse intervals were 30 s.

To elicit gastric mill rhythms in decentralized nervous system preparations, we extracellularly stimulated the axon of MCN1 in the part of the transected *ion* that remained connected to the STG (Fig 1A). This reliably and specifically elicited a specific version of the gastric mill rhythm at 10°C. This version of the rhythm has been characterized in detail before [11,12,25]. Both *ions* were tonically stimulated for 200 s with the same frequency (Master-8 stimulator [AMPI], 1 ms pulse duration). The *ion* contains the axons of two projection neurons (MCN1 and MCN5). The extracellular activation threshold is lower for MCN1 than for MCN5 [12,53]. In all experiments, we confirmed that MCN1 was selectively activated by adjusting MCN1 activation threshold separately for each *ion* by slowly increasing stimulation voltage until a PSP in LG was obtained (for details, see [12]). In addition, we monitored the activity of the pyloric LP neuron to confirm whether MCN5 had been activated or not. LP is strongly inhibited by MCN5 [56], and activation of this projection neuron results in a prominent decrease in LP firing frequency. Preparations without discrete activation thresholds for MCN1 and MCN5 were discarded and not used for experiments. The presence of LP PSPs during *ion* stimulation was also used to confirm that MCN1 activation threshold was still effective at elevated temperatures. Stimulation frequency was increased in 1 Hz steps until a gastric mill rhythm could be observed at 10°C (= “threshold frequency”).

In vitro preparations were continuously superfused with physiological saline. To manipulate temperature of the STG, we built a petroleum jelly well around the STG to thermally isolate it from the rest of the nervous system. Temperature inside and outside of the well was controlled independently with two saline superfusion lines, cooled by separate Peltier devices. Temperature was continuously measured close to the STG and CoGs with separate temperature probes (Voltcraft 300K). We selectively altered temperature at the STG between 10°C and 13°C, while the surrounding nervous system was kept constantly at 10°C. In experiments in which the STG was decentralized by transecting *ion*s and *son*s, the temperature of the whole bath was changed. To evaluate temperature effects on MCN1 activity (Fig 6A), the temperature at the saline surrounding the CoGs was varied. In this case, the CoGs were isolated from the STG by transecting all connecting nerves. MCN1 action potentials were recorded extracellularly from the *ion* stump still connected to the CoG. The temperature was changed by ~1°C/min unless otherwise mentioned. With intracellular recordings, the temperature was changed by ~1°C/6–7 min to prevent swelling of the neurons. Measurements were taken after 10 min at the target temperature.

Dynamic clamp [28] was used to inject artificial leak currents into LG using Spike2 software and two electrode current clamp mode. In each preparation, input resistance and resting potential were measured at 10°C and 13°C to determine temperature-induced changes. Leak conductance was calculated from these measurements and computed in dynamic clamp according to the following:

$$I_{dyn}=\Delta leak*(V-E),$$

where *I*_*dyn*_ is the injected current, *Δleak* represents the difference in leak conductance between 10°C and 13°C, and *V* the membrane potential. *E* was taken as the resting potential at 13°C. *E* and *Δleak* were calculated separately for each preparation.

### Computer Models

The effects of leak and I_MI_ on pattern generating networks were modeled with *MadSim* [36,57] (freely available for download at http://www.neurobiologie.de and added as supplemental S1 Data) using standard morphology and passive properties according to [58]. Active membrane properties were implemented according to modified Hodgkin-Huxley equations [59,60]. Leak was implemented as instantaneous linear current in the form of

I=g*(V−E),

with g being the maximum conductance, *V* the membrane potential, and *E* the reversal potential. The reversal potential was set to resting potential in all simulations, and *g* was varied. I_MI_ was implemented as noninactivating current using

$$I=\bar{g}*a^{p}*(V-E),$$

with *p* = 1, *E* = 0, and varying $\bar{g}$. Activation a was calculated using

$$a=\frac{1}{1+e^{\frac{V-V_{0}}{s}}},$$

with *V*_0_ = −40 mV and *s* = −10 mV. Maximum conductance $\bar{g}$ was varied. The time constant of activation was set to 50 ms in all models. The model contained the core gastric mill and pyloric networks and was built with MCN1, LG, Int1, and AB according to the real network configuration [7]. The MCN1 terminal was modeled as a separated compartment as it receives synaptic feedback from LG [12]. MCN1 was activated with 15 Hz current pulses. AB membrane potential oscillations were elicited with a sinusoidal current of 1 Hz to provide pyloric feedback to Int1. We used this model to run an exhaustive search with 1,100 simulations by altering leak conductance ($g_{leak}$) and I_MI_ conductance (${\bar{g}}_{IMI}$) of LG. All other neurons and parameters were left unchanged. *g*_*leak*_ and ${\bar{g}}_{IMI}$ were increased linearly within physiologically realistic values ($g_{leak}=\;1$ to 106 nS, 22 steps, 5 nS step size; ${\bar{g}}_{IMI}$ = 1 to 246 nS, 50 steps, 5 nS step size). For all models, simulations produced 100 s-long voltage waveforms. Kinetic parameters for the ionic conductances were set to physiologically realistic values. Maximum conductances $\bar{g}$ of ionic conductances in the model neurons were chosen to achieve a functional (network) output. The model and the simulation are provided as S1 Data and can be found at ModelDB database (Accession Number 184404).

### Data Analysis

The gastric mill rhythm was considered active when LG produced regular bursts in alternation with the DG neuron. Cycle period was defined as the time between the onset of an LG burst and the onset of the next burst. Rhythmicity was defined as a series of busts with interburst intervals of at least 2 s. Bursts were defined as a series of at least four action potentials with interspike intervals (ISIs) below 1 s. We refer to termination of the rhythm if either one or all of those criteria were not fulfilled. Pyloric cycle period was determined using PD bursts. Intraburst firing frequency was defined as the number of spikes within a burst minus one divided by burst duration. Mean values were determined from at least ten consecutive cycles of pyloric activity and phase as normalized time during a cycle.

Figures were prepared with CorelDraw X3 (Corel Cooperation), Excel 2010, SigmaStat, and SigmaPlot (version 11, Jandel Scientific). Color maps were generated with Spike2. Data used for analyses and figure generation are given as S2 Data. Unless stated otherwise, data are presented as mean ± SD when normally distributed or as box plot (25% and 75% quartiles plus fifth/95th percentile, lines = median, diamonds = mean) for nonparametric data. Alternatively, individual data points for each animal are given. Significant differences are stated as * *p* < 0.05, ** *p* < 0.01, *** *p* < 0.001.

## Supporting Information

S1 Data Model and model environment. (ZIP)

S2 Data Data for all figures. (XLSX)

S1 Fig The phase relationship of the pyloric rhythm is resilient against temperature perturbations and independent from upstream modulatory projection neuron activity. (A) Example extracellular nerve recordings showing the triphasic pyloric rhythm at 10°C (top) and 13°C (bottom) without spontaneous gastric mill rhythm. Top: pyloric dilator nerve *pdn* showing the sole activity of the PD neurons. Bottom: lateral ventricular nerve *lvn* showing the triphasic rhythm with PD, LP, and PY activities. At 13°C, the cycle period decreased substantially, but the rhythmicity and relative timing of the triphasic pattern were largely preserved. (B–E) Quantification of pyloric network output at 10°C (blue) and 13°C (red). (B) On average, the pyloric cycle period decreases significantly at 13°C. Paired *t*-test, *p* < 0.001. (C) The phase relationship and (D) the number of spikes/burst of PD, LP, and PY were maintained at different temperatures. (E) The intraburst spike frequency of LP and PY increased significantly at 13°C. Paired *t*-test, *p* < 0.05.
(TIF)

## Abbreviations

AB: anterior burster
AP: action potential
CabTRP Ia: *Cancer borealis* tachykinin-related peptide Ia
CoG: commissural ganglion
CPG: central pattern generator
DG: dorsal gastric
dgn: dorsal gastric nerve
ePSP: electrical postsynaptic potential
I_MI_: modulator-induced current
Int1: Interneuron 1
*ion*: inferior esophageal nerve
ISI: interspike interval
LG: lateral gastric
*lgn*: lateral gastric nerve
LP: lateral pyloric
*lvn*: lateral ventricular nerve
MCN: modulatory commissural neuron
NMDA: N-Methyl-D-aspartate
PD: pyloric dilator
*pdn*: pyloric dilator nerve
PY: pyloric constrictor
RM ANOVA: repeated measures analysis of variance
SD: standard deviation
SEM: standard error of the mean
*son*: superior esophageal nerve
STG: stomatogastric ganglion
*stn*: stomatogastric nerve
STNS: stomatogastric nervous system
TTX: tetrodotoxin
