# Supplementary material for: Neuromodulation to the Rescue: Compensation of Temperature-Induced Breakdown of Rhythmic Motor Patterns via Extrinsic Neuromodulatory Input
Source: PLoS Biol. 2015 Sep 29;13(9):e1002265. doi: 10.1371/journal.pbio.1002265 (PMC4587842; doi:10.1371/journal.pbio.1002265)
Supplement: S1 Data — (ZIP) [file pbio.1002265.s001.zip › S1_Data_Instruction.pdf]

Instructions on how to use the model environment MadSim to study the interaction of leak and I_{MI} on pattern generating networks

S1 Data is a ZIP folder containing a Hodgkin-Huxley based circuit model and the simulation environment used for the data shown in the paper: “Neuromodulation to the rescue: compensation of temperature-induced breakdown of rhythmic motor patterns via extrinsic neuromodulatory input” by C. Städele, S. Heigele and W. Stein. MadSim, the simulation environment used for this study, is freeware and included in the package. MadSim was developed by Dr. Wolfgang Mader (Institute of Neurobiology, Ulm University, Germany). Dr. Mader is continuously improving the program, including translation into English. Some commands may appear in German. The programmer can be contacted at wolfgang.mader@uni-ulm.de.

The ZIP file contains:

1. The simulation environment MadSim (ver 6.11) for Windows. MadSim has previously used for modeling of neurons and networks [1-3].
2. Two simulation files:
    1) *S1_Data_model.gui* (balanced leak and I_{MI} conductances, LG neuron is rhythmically active)
    2) *S1_Data_model_high_leak.gui* (unbalanced leak and I_{MI} conductances, LG is silent)
3. This document

To use:

a) Extract the files and folders contained in the ZIP file to a folder of your choice (e.g. Desktop)
b) Go to the MadSim 6.11 folder (Fig. 1) and double click on **“madsim.exe”**. MadSim runs without installation.
c) The prompt will ask you to open a simulation file. To open the model, select the ***S1_Data_model.gui*** in the folder into which you extracted the ZIP file.
d) You will now see the network structure and an empty result window for the LG neuron (Fig. 2). The network consists of modulatory projection neuron MCN1, a representation of its axon terminal (term; a separate compartment), the gastric mill half-center neurons LG and Int1, plus a representation of the pyloric rhythm (AB). For details see Fig. 1 of the main text. MCN1 and AB are driven by somatic current injections. All neurons are Hodgkin-Huxley type neurons (see manuscript text).
e) Press F2 to start the simulation. The LG membrane potential will be displayed in the LG result window (Fig. 3 left). Note that the membrane potential of every neuron (or even the whole network) can be visualized by selecting one or several neurons and opening result views (CTRL plus M).
f) Please be patient as the calculations may require several dozen seconds. Calculation speed depends on many factors, including the step width of the simulation (0.02 ms) and your computer processing speed. The model simulation time is preset to 100s. To accelerate the calculation, simulation time can be reduced. To do this click on the ‘Settings’ tab and select ‘simulation settings’.
g) The main point of this model is to study the effects of interactions between the CabTRP-induced ionic current (I_{MI}) and the leak current. The leak current changes with temperature (see main text). The hypothesis tested is whether I_{MI} acts as a negative leak and counterbalances the temperature-induced increase of the leak. For this, leak and I_{MI} can be changed independently. To view or change the model parameters right click on the neuron of choice (for example LG) and select ***‘neuron’*** and ***‘physiological parameters’*** (for leak) or ***‘neuron***’, ***‘channel definitions’*** and ‘***userdefined channels***’ (for the CabTRP induced I_{MI}, see Fig. 3 right). A new window will open showing the channel settings for leak or I_{MI}.

h) We used this model to run an exhaustive search with 1,100 simulations. gleak and $\bar{g}l_{MI}$ were increased linearly (gleak=1 to 106 nS, 22 steps, 5 nS step size; $\bar{g}l_{MI}$=1 to 246 nS, 50 steps, 5 nS step size). Simulations were always 100 s long. Kinetic parameters for the ionic conductances in all neurons were set to physiologically realistic values. Maximum conductances $\bar{g}$ of ionic conductances in the model neurons were chosen to achieve a functional (network) output.

i) To change gleak, open the leak settings window as described above. To change conductance levels, edit the values next to ***'conductance (membrane leakage)'*** for both the ***'soma parameters'*** and the ***'dendritic parameters'***. In all of our simulations, leak conductance for the soma and dendrites was identical.

j) For demonstration, we provide two simulation files: Supplemental 2_model.gui, which shows the normal burst activity of the LG neuron and *S1_Data_model_high_leak.gui*, which shows that the rhythm stops when the leak current is increased by a factor of 2.

k) gui simulation files are ASCII files and can be edited by experienced users.

Fig. 1. MadSim folder after extracting the Zip file. The Madsim.exe is highlighted.

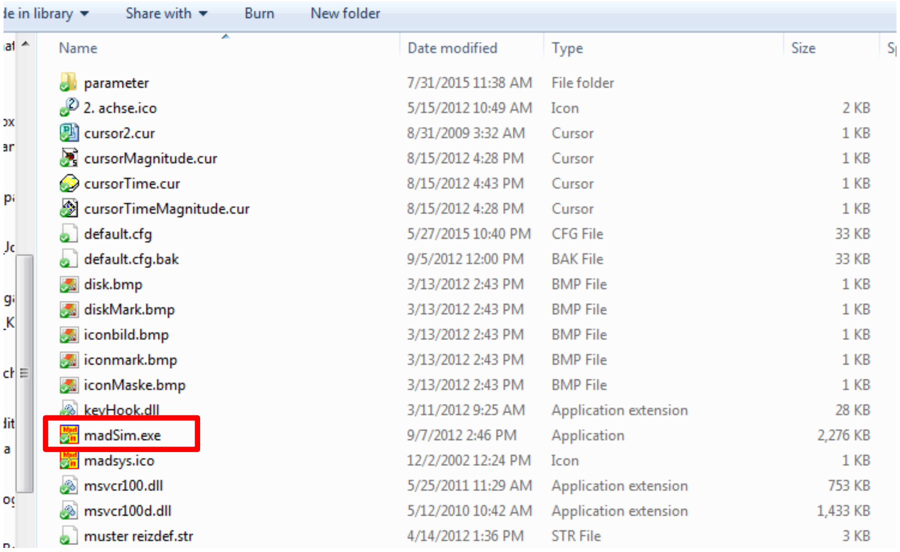

Fig. 2. The Madsim user interface after opening the simulation file 'S1_Data_model.gui'.

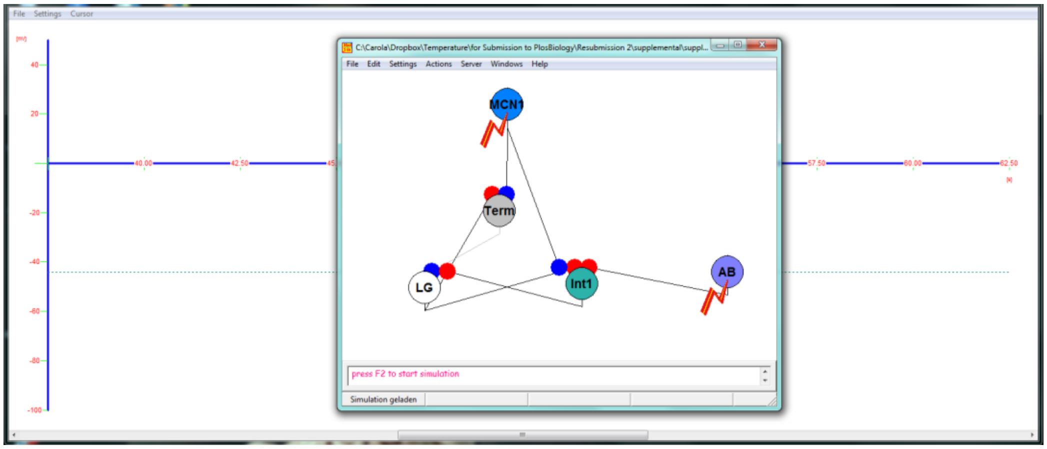

Fig. 3. Left: LG membrane potential with a $\bar{g}(I_{MI})$ of 70 nS and a $\bar{g}$(leak) of 21 nS. Right: MadSim user menu for editing ionic conductances.

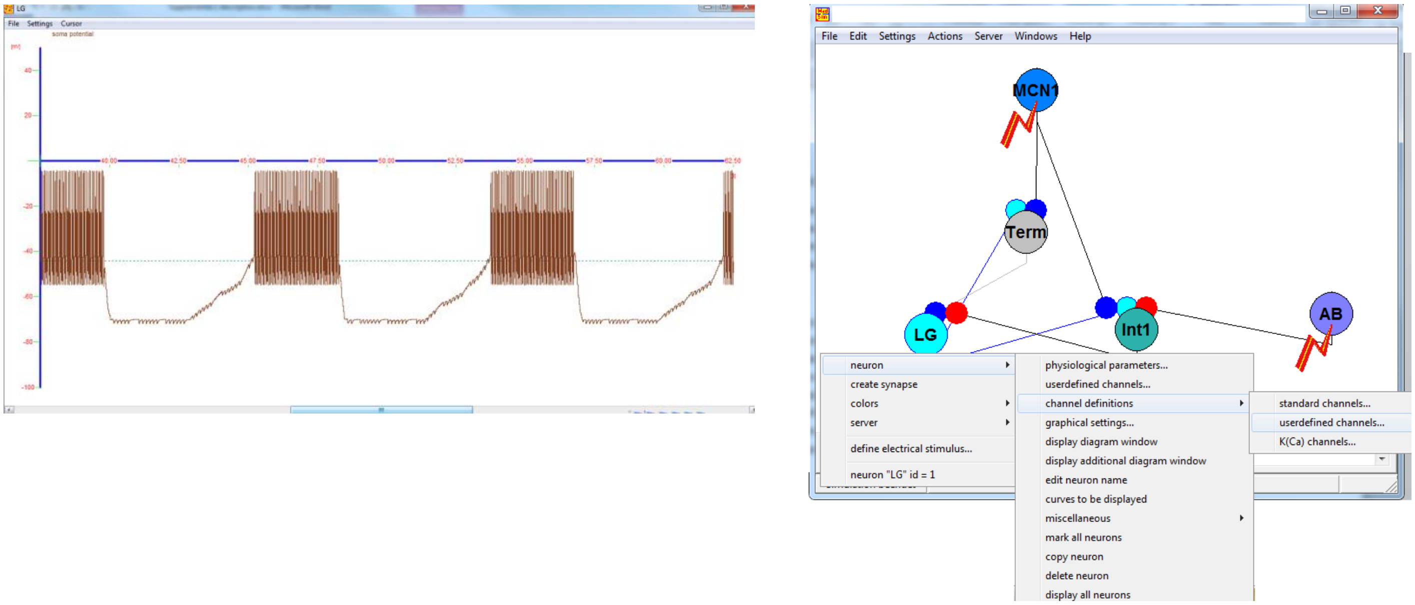

References for the MadSim simulation environment:

[1] Stein W, Straub O, Ausborn J, Mader W, Wolf H. Motor pattern selection by combinatorial code of interneuronal pathways. J Comput Neurosci. 2008; 25: 543-561.

[2] Ausborn J, Stein W, Wolf H. Frequency Control of Motor Patterning by Negative Sensory Feedback. J Neurosci. 2007; 27: 9319-9328.

[3] Daur N, Diehl F, Mader W, Stein W. The stomatogastric nervous system as a model for studying sensorimotor interactions in real-time closed-loop conditions. Front Comput Neurosci. 2012; 6.
