# Supplementary figures and images for: Neuromodulation to the Rescue: Compensation of Temperature-Induced Breakdown of Rhythmic Motor Patterns via Extrinsic Neuromodulatory Input
Source: PLoS Biol. 2015 Sep 29;13(9):e1002265. doi: 10.1371/journal.pbio.1002265 (PMC4587842; doi:10.1371/journal.pbio.1002265)

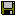

Supplement: S1 Data — (ZIP) [file pbio.1002265.s001.zip › madSim 6.11/disk.bmp]

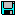

Supplement: S1 Data — (ZIP) [file pbio.1002265.s001.zip › madSim 6.11/diskMark.bmp]

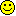

Supplement: S1 Data — (ZIP) [file pbio.1002265.s001.zip › madSim 6.11/iconbild.bmp]

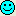

Supplement: S1 Data — (ZIP) [file pbio.1002265.s001.zip › madSim 6.11/iconmark.bmp]

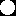

Supplement: S1 Data — (ZIP) [file pbio.1002265.s001.zip › madSim 6.11/iconMaske.bmp]

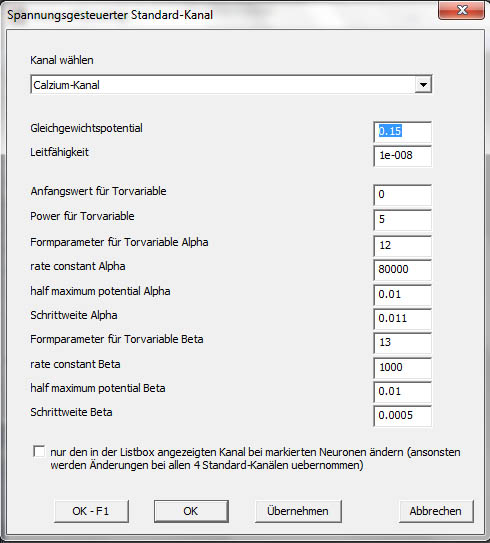

Supplement: S1 Data — (ZIP) [file pbio.1002265.s001.zip › madSim 6.11/parameter/standard-kanaele/ca-kanal.jpg]

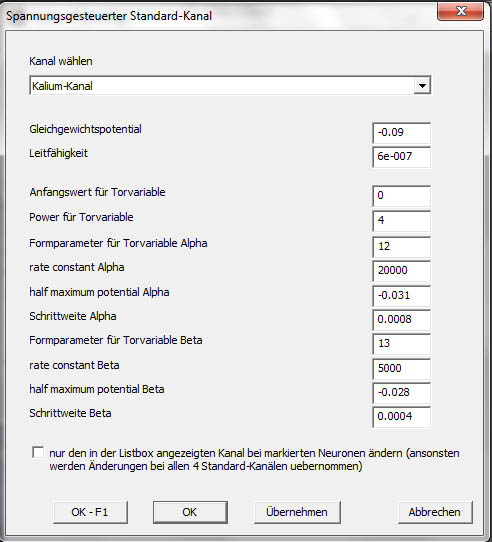

Supplement: S1 Data — (ZIP) [file pbio.1002265.s001.zip › madSim 6.11/parameter/standard-kanaele/k-kanal.jpg]

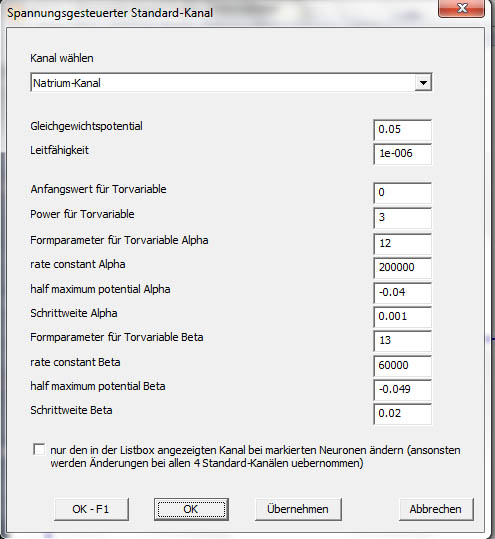

Supplement: S1 Data — (ZIP) [file pbio.1002265.s001.zip › madSim 6.11/parameter/standard-kanaele/na-kanal aktivierung.jpg]

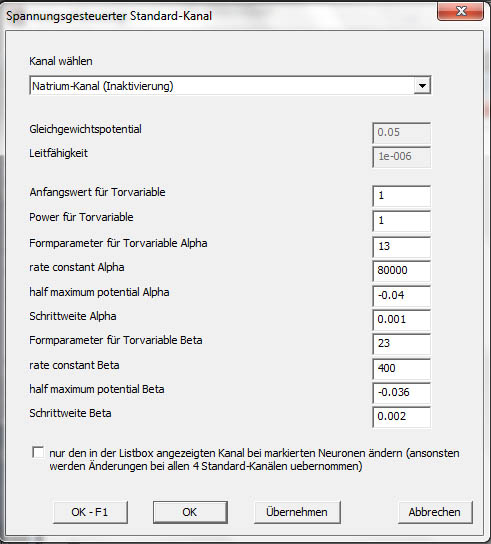

Supplement: S1 Data — (ZIP) [file pbio.1002265.s001.zip › madSim 6.11/parameter/standard-kanaele/na-kanal inaktivierung.jpg]

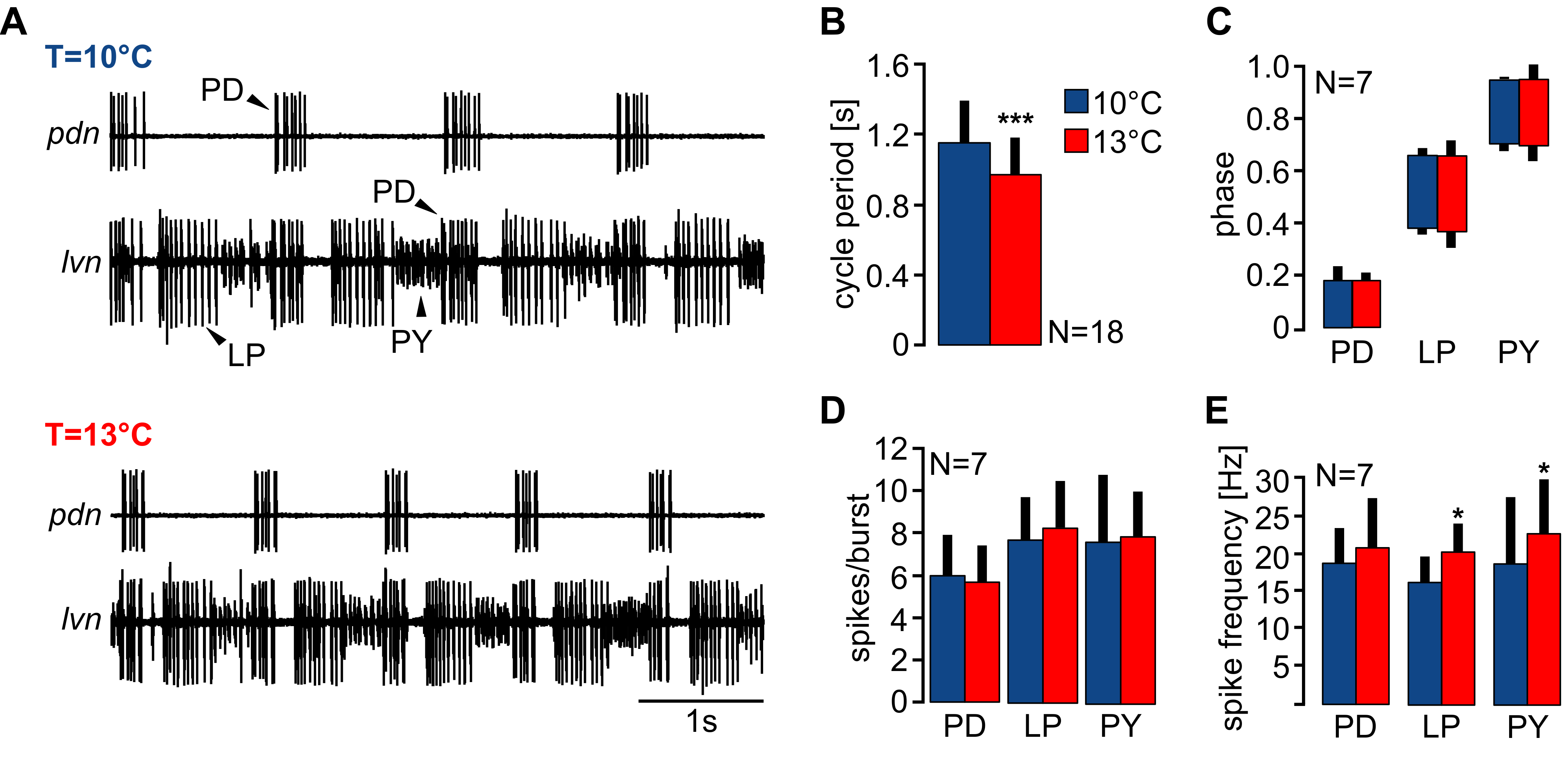

Supplement: S1 Fig — (A) Example extracellular nerve recordings showing the triphasic pyloric rhythm at 10°C (top) and 13°C (bottom) without spontaneous gastric mill rhythm. Top: pyloric dilator nerve pdn showing the sole activity of the PD neurons. Bottom: lateral ventricular nerve lvn showing the triphasic rhythm with PD, LP, and PY activities. At 13°C, the cycle period decreased substantially, but the rhythmicity and relative timing of the triphasic pattern were largely preserved. (B–E) Quantification of pyloric network output at 10°C (blue) and 13°C (red). (B) On average, the pyloric cycle period decreases significantly at 13°C. Paired t-test, p < 0.001. (C) The phase relationship and (D) the number of spikes/burst of PD, LP, and PY were maintained at different temperatures. (E) The intraburst spike frequency of LP and PY increased significantly at 13°C. Paired t-test, p < 0.05. (TIF) [file pbio.1002265.s003.tif]
