# Supplementary material for: Neuromodulation to the Rescue: Compensation of Temperature-Induced Breakdown of Rhythmic Motor Patterns via Extrinsic Neuromodulatory Input
Source: PLoS Biol. 2015 Sep 29;13(9):e1002265. doi: 10.1371/journal.pbio.1002265 (PMC4587842; doi:10.1371/journal.pbio.1002265)
Supplement: S1 Data — (ZIP) [file pbio.1002265.s001.zip › madSim 6.11/parameter/GB.channel.description.doc]

GB_CA_INITIAL	- initial calcium concentration

GB_K_ICA	- cell volume dependend, formula: (1/(2*Faraday-constant*cell_volume))

GB_K_CA	- speed of calcium restitution

GB_C0_CA	- constant background concentration of calcium

GB_CA_CA	- extracellular calcium concentration

GB_TEMP	- absolute temperature

GB_M_POWER	- power of gate variable m

GB_M_INITIAL	- initial value of gate variable m

GB_H_POWER	- power of gate variable h

GB_H_INITIAL	- initial value of gate variable h

GB_NA_G	- conductance of sodium channel

GB_NA_E	- equilibrium potential of sodium channel

GB_Form_Alpha_M	- form parameter for gate variable m, alpha term

GB_AalphaM	- rate constant

GB_V0alphaM	- half maximum potential

GB_BalphaM	- step width

GB_Form_Beta_M	- form parameter for gate variable m, beta term

GB_AbetaM	- rate constant

GB_V0betaM	- half maximum potential

GB_BbetaM	- step width

GB_Form_Alpha_H	- form parameter for gate variable h, alpha term

GB_AalphaH	- rate constant

GB_V0alphaH	- half maximum potential

GB_BalphaH	- step width

GB_Form_Beta_H	- form parameter for gate variable h, beta term

GB_AbetaH	- rate constant

GB_V0betaH	- half maximum potential

GB_BbetaH	- step width

GB_N_POWER	- power of gate variable n

GB_N_INITIAL	- initial value of gate variable n

GB_K_G	- conductance of potassium channel

GB_K_E	- equilibrium potential of potassium channel

GB_Form_Alpha_N	- form parameter for gate variable n, alpha term

GB_AinfinityN	- rate constant

GB_V0infinityN	- half maximum potential

GB_BinfinityN	- step width

GB_Form_Beta_N	- form parameter for gate variable n, beta term

GB_AtauN	- rate constant

GB_V0tauN	- half maximum potential

GB_BtauN	- step width

GB_A_POWER	- power of gate variable a

GB_A_INITIAL	- initial value of gate variable a

GB_B1_POWER	- power of gate variable beta 1

GB_B1_INITIAL	- initial value of gate variable beta 1

GB_B2_POWER	- power of gate variable beta 2

GB_B2_INITIAL	- initial value of gate variable beta 2

GB_G_POWER	- power of gate variable gamma

GB_G_INITIAL	- initial value of gate variable gamma

GB_A_G	- conductance of A-channel

GB_A_E	- equilibrium potential of A-channel

GB_Form_Alpha_A	- form parameter for gate variable a, alpha term

GB_V0a	- half maximum potential

GB_Ba	- step width

GB_Form_Beta_A	- form parameter for gate variable a, beta term

GB_Ta	- time constant (= 1 / rate constant)

GB_Form_Alpha_B1	- form parameter for gate variable b1, alpha term

GB_V0b1	- half maximum potential

GB_Bb1	- step width

GB_Form_Beta_B1	- form parameter for gate variable b1, beta term

GB_Tb1	- time constant (= 1 / rate constant)

GB_Form_Alpha_B2	- form parameter for gate variable b2, alpha term

GB_V0alphab2	- half maximum potential

GB_Balphab2	- step width

GB_Form_Beta_B2	- form parameter for gate variable b2, beta term

GB_Ab2	- rate constant for gate variable b2

GB_V0b2	- half maximum potential

GB_Bb2	- step width

GB_V0g	- half maximum potential for gate variable gamma

GB_Bg	- step width for gate variable gamma

GB_C_POWER	- power of gate variable c

GB_C_INITIAL	- initial value of gate variable c

GB_D_POWER	- power of gate variable d

GB_D_INITIAL	- initial value of gate variable d

GB_CA1_G	- conductance of first calcium channel

GB_Form_Alpha_C	- form parameter for gate variable c, alpha term

GB_V0c	- half maximum potential

GB_Bc	- step width

GB_Form_Beta_C	- form parameter for gate variable c, beta term

GB_Tc	- time constant (= 1 / rate constant)

GB_Form_Alpha_D	- form parameter for gate variable d, alpha term

GB_V0d	- half maximum potential

GB_Bd	- step width

GB_Form_Beta_D	- form parameter for gate variable d, beta term

GB_Td	- time constant (= 1 / rate constant)

GB_E_POWER	- power of gate variable e

GB_E_INITIAL	- initial value of gate variable e

GB_CA2_G	- conductance of second calcium channel

GB_Form_Alpha_E	- form parameter for gate variable e, alpha term

GB_V0e	- half maximum potential

GB_Be	- step width

GB_Form_Beta_E	- form parameter for gate variable e, beta term

GB_Te	- time constant (= 1 / rate constant)

GB_P_POWER	- power of gate variable p

GB_P_INITIAL	- initial value of gate variable p

GB_Q_POWER	- power of gate variable q

GB_Q_INITIAL	- initial value of gate variable q

GB_KCA_G	- conductance of calcium dependend potassium channel

GB_KCA_E	- equilibrium potential of calcium dependend potassium channel

GB_Form_Alpha_P	- form parameter for gate variable p, alpha term

GB_V0p1	- half maximum potential

GB_Bp1	- step width

GB_Form_Beta_P	- form parameter for gate variable p, beta term

GB_V0p2	- half maximum potential

GB_Bp2	- step width

GB_Tp	- time constant (= 1 / rate constant)

GB_Cca	- speed of calcium restitution for gate variable p

GB_C1	- calcium concentration constant for gate variable p

GB_Form_Alpha_Q	- form parameter for gate variable q, alpha term

GB_C2	- calcium concentration constant for gate variable q

GB_C3	- calcium concentration constant for gate variable q

GB_Form_Beta_Q	- form parameter for gate variable q, beta term

GB_Tq	- time constant (= 1 / rate constant)

GB_R_POWER	- power of gate variable r

GB_R_INITIAL	- initial value of gate variable r

GB_IR_G	- conductance of IR-channel

GB_IR_E	- equilibrium potential of IR-channel

GB_Form_Alpha_R	- form parameter for gate variable r, alpha term

GB_V0r	- half maximum potential

GB_Br	- step width

GB_Form_Beta_R	- form parameter for gate variable r, beta term

GB_Atau_r	- time constant (= 1 / rate constant)

GB_V0tau_r	- half maximum potential

GB_Btau_r	- step width

GB_S_POWER	- power of gate variable n

GB_S_INITIAL	- initial value of gate variable n

GB_P_G	- conductance of potassium channel

GB_P_E	- equilibrium potential of potassium channel

GB_Form_Alpha_S	- form parameter for gate variable s, alpha term

GB_V0s	- half maximum potential

GB_Bs	- step width

GB_Form_Beta_S	- form parameter for gate variable s, beta term

GB_Ts	- time constant (= 1 / rate constant)

GB_SPIKE_THRESHOLD	- spike threshold, used only for spike identification
