# Supplementary material for: Neuromodulation to the Rescue: Compensation of Temperature-Induced Breakdown of Rhythmic Motor Patterns via Extrinsic Neuromodulatory Input
Source: PLoS Biol. 2015 Sep 29;13(9):e1002265. doi: 10.1371/journal.pbio.1002265 (PMC4587842; doi:10.1371/journal.pbio.1002265)
Supplement: S1 Data — (ZIP) [file pbio.1002265.s001.zip › madSim 6.11/parameter/HH.channel.description.doc]

```
/************************************************************************************************************************

	PROGRAM:	BIOSIM

	FILENAME:	HH.channel.description

	PURPOSE:	description of all parameters used in the HH.channel file

************************************************************************************************************************/

HH_NA_E	- equilibrium potential of sodium channel
HH_NA_G	- conductance of sodium channel
HH_K_E	- equilibrium potential of potassium channel
HH_K_G	- conductance of potassium channel
HH_Form_Alpha_M	- form parameter for gate variable m, alpha term
HH_AalphaM	- rate constant
HH_V0alphaM	- half maximum potential
HH_BalphaM	- step width
HH_Form_Beta_M	- form parameter for gate variable m, beta term
HH_AbetaM	- rate constant
HH_V0betaM	- half maximum potential
HH_BbetaM	- step width
HH_M_POWER	- power of gate variable m
HH_M_INITIAL	- initial value of gate variable m
HH_Form_Alpha_H	- form parameter for gate variable h, alpha term
HH_AalphaH	- rate constant
HH_V0alphaH	- half maximum potential
HH_BalphaH	- step width
HH_Form_Beta_H	- form parameter for gate variable h, beta term
HH_AbetaH	- rate constant
HH_V0betaH	- half maximum potential
HH_BbetaH	- step width
HH_H_POWER	- power of gate variable h
HH_H_INITIAL	- initial value of gate variable h
HH_Form_Alpha_N	- form parameter for gate variable n, alpha term
HH_AalphaN	- rate constant
HH_V0alphaN	- half maximum potential
HH_BalphaN	- step width
HH_Form_Beta_N	- form parameter for gate variable n, beta term
HH_AbetaN	- rate constant
HH_V0betaN	- half maximum potential
HH_BbetaN	- step width
HH_N_POWER	- power of gate variable n
HH_N_INITIAL	- initial value of gate variable n
HH_SPIKE_THRESHOLD	- spike threshold, used only for spike identification
```
