# Supplementary material for: Neuromodulation to the Rescue: Compensation of Temperature-Induced Breakdown of Rhythmic Motor Patterns via Extrinsic Neuromodulatory Input
Source: PLoS Biol. 2015 Sep 29;13(9):e1002265. doi: 10.1371/journal.pbio.1002265 (PMC4587842; doi:10.1371/journal.pbio.1002265)
Supplement: S1 Data — (ZIP) [file pbio.1002265.s001.zip › madSim 6.11/parameter/ONOFF.channel.description.doc]

```
/************************************************************************************************************************

	PROGRAM:	BIOSIM

	FILENAME:	ONOFF.channel.description

	PURPOSE:	description of all parameters used in the ONOFF.channel file


************************************************************************************************************************/

ONOFF_NA_E	- equilibrium potential of sodium channel
ONOFF_NA_CONDUCTANCE	- conductance of sodium channel
ONOFF_NA_DURATION	- opening duration of sodium channel
ONOFF_K_E	- equilibrium potential of potassium channel
ONOFF_K_CONDUCTANCE	- conductance of potassium channel
ONOFF_K_START	- start time of potassium channel opening after threshold
ONOFF_K_DURATION	- opening duration of potassium channel
ONOFF_CA_E	- equilibrium potential of calcium channel
ONOFF_CA_CONDUCTANCE	- conductance of calcium channel
ONOFF_CA_START	- start time of calcium channel opening after threshold
ONOFF_CA_DURATION	- opening duration of calcium channel
ONOFF_SPIKE_THRESHOLD	- spike threshold
```
