# Supplementary material for: Neuromodulation to the Rescue: Compensation of Temperature-Induced Breakdown of Rhythmic Motor Patterns via Extrinsic Neuromodulatory Input
Source: PLoS Biol. 2015 Sep 29;13(9):e1002265. doi: 10.1371/journal.pbio.1002265 (PMC4587842; doi:10.1371/journal.pbio.1002265)
Supplement: S1 Data — (ZIP) [file pbio.1002265.s001.zip › madSim 6.11/parameter/SWIM.channel.description.doc]

```
/************************************************************************************************************************

	PROGRAM:	BIOSIM

	FILENAME:	SWIM.channel.description

	PURPOSE:	description of all parameters used in the SWIM.channel file


************************************************************************************************************************/

SWIM_NA_E	- equilibrium potential of sodium channel
SWIM_NA_G	- conductance of sodium channel
SWIM_Form_Alpha_M	- form parameter for gate variable m, alpha term
SWIM_AalphaM	- rate constant
SWIM_V0alphaM	- half maximum potential
SWIM_BalphaM	- step width
SWIM_Form_Beta_M 	- form parameter for gate variable m, beta term
SWIM_AbetaM	- rate constant
SWIM_V0betaM	- half maximum potential
SWIM_BbetaM	- step width
SWIM_M_POWER	- power of gate variable m
SWIM_M_INITIAL	- initial value of gate variable m
SWIM_Form_Alpha_H	- form parameter for gate variable h, alpha term
SWIM_AalphaH	- rate constant
SWIM_V0alphaH	- half maximum potential
SWIM_BalphaH	- step width
SWIM_Form_Beta_H	- form parameter for gate variable h, beta term
SWIM_AbetaH	- substitudes complete linoid term
SWIM_V0betaH	- half maximum potential
SWIM_BbetaH	- step width
SWIM_H_POWER	- power of gate variable h
SWIM_H_INITIAL	- initial value of gate variable h
SWIM_K_E	- equilibrium potential of potassium channel
SWIM_K_G	- conductance of potassium channel
SWIM_Form_Alpha_N	- form parameter for gate variable n, alpha term
SWIM_AalphaN	- rate constant
SWIM_V0alphaN	- half maximum potential
SWIM_BalphaN	- step width
SWIM_Form_Beta_N	- form parameter for gate variable n, beta term
SWIM_AbetaN	- rate constant
SWIM_V0betaN	- half maximum potential
SWIM_BbetaN	- step width
SWIM_N_POWER	- power of gate variable n
SWIM_N_INITIAL	- initial value of gate variable n
SWIM_CA_E	- equilibrium potential of calcium channel
SWIM_CA_G	- conductance of calcium channel
SWIM_Form_Alpha_C	- form parameter for gate variable c, alpha term
SWIM_AalphaC	- rate constant
SWIM_V0alphaC	- half maximum potential
SWIM_BalphaC	- step width
SWIM_Form_Beta_C	- form parameter for gate variable c, beta term
SWIM_AbetaC	- rate constant
SWIM_V0betaC	- half maximum potential
SWIM_BbetaC	- step width
SWIM_C_POWER	- power of gate variable c
SWIM_C_INITIAL	- initial value of gate variable c
SWIM_SPIKE_THRESHOLD	- spike threshold, used only for spike identification
```
